# Canagliflozin mediates tumor suppression alone and in combination with radiotherapy in non‐small cell lung cancer (NSCLC) through inhibition of HIF‐1α

**DOI:** 10.1002/1878-0261.13508

**Published:** 2023-08-27

**Authors:** Olga‐Demetra Biziotis, Evangelia Evelyn Tsakiridis, Amr Ali, Elham Ahmadi, Jianhan Wu, Simon Wang, Bassem Mekhaeil, Kanwaldeep Singh, Gabe Menjolian, Thomas Farrell, Bassam Abdulkarim, Ranjan K. Sur, Aruz Mesci, Peter Ellis, Tobias Berg, Jonathan L Bramson, Paola Muti, Gregory R Steinberg, Theodoros Tsakiridis

**Affiliations:** ^1^ Centre for Metabolism, Obesity and Diabetes Research McMaster University Hamilton Canada; ^2^ Centre for Discovery in Cancer Research McMaster University Hamilton Canada; ^3^ Department of Oncology McMaster University Hamilton Canada; ^4^ Department of Medicine McMaster University Hamilton Canada; ^5^ Radiotherapy Program Juravinski Cancer Centre Hamilton Canada; ^6^ Radiation Physics Program Juravinski Cancer Centre Hamilton Canada; ^7^ Department of Oncology McGill University Montréal Canada; ^8^ Division of Radiation Oncology Juravinski Cancer Centre Hamilton Canada; ^9^ Department of Pathology and Molecular Medicine McMaster University Hamilton Canada; ^10^ Michael DeGroote Institute for Infectious Disease Research McMaster University Hamilton Canada; ^11^ Department of Biomedical, Surgical and Dental Sciences University of Milan Italy; ^12^ Department of Biochemistry and Biomedical Sciences McMaster University Hamilton Canada

**Keywords:** canagliflozin, HIF‐1α, lung cancer, mTOR, radiotherapy

## Abstract

Non‐small cell lung cancer (NSCLC) has a poor prognosis, and effective therapeutic strategies are lacking. The diabetes drug canagliflozin inhibits NSCLC cell proliferation and the mammalian target of rapamycin (mTOR) pathway, which mediates cell growth and survival, but it is unclear whether this drug can enhance response rates when combined with cytotoxic therapy. Here, we evaluated the effects of canagliflozin on human NSCLC response to cytotoxic therapy in tissue cultures and xenografts. Ribonucleic acid sequencing (RNA‐seq), real‐time quantitative PCR (RT‐qPCR), metabolic function, small interfering ribonucleic acid (siRNA) knockdown, and protein expression assays were used in mechanistic analyses. We found that canagliflozin inhibited proliferation and clonogenic survival of NSCLC cells and augmented the efficacy of radiotherapy to mediate these effects and inhibit NSCLC xenograft growth. Canagliflozin treatment alone moderately inhibited mitochondrial oxidative phosphorylation and exhibited greater antiproliferative capacity than specific mitochondrial complex‐I inhibitors. The treatment downregulated genes mediating hypoxia‐inducible factor (HIF)‐1α stability, metabolism and survival, activated adenosine monophosphate‐activated protein kinase (AMPK) and inhibited mTOR, a critical activator of hypoxia‐inducible factor‐1α (HIF‐1α) signaling. HIF‐1α knockdown and stabilization experiments suggested that canagliflozin mediates antiproliferative effects, in part, through suppression of HIF‐1α. Transcriptional regulatory network analysis pinpointed histone deacetylase 2 (*HDAC2*), a gene suppressed by canagliflozin, as a key mediator of canagliflozin's transcriptional reprogramming. *HDAC2* knockdown eliminated HIF‐1α levels and enhanced the antiproliferative effects of canagliflozin. HDAC2‐regulated genes suppressed by canagliflozin are associated with poor prognosis in several clinical NSCLC datasets. In addition, we include evidence that canagliflozin also improves NSCLC response to chemotherapy. In summary, canagliflozin may be a promising therapy to develop in combination with cytotoxic therapy in NSCLC.

## Graphical abstract

The diabetes drug canagliflozin suppresses non‐small cell lung cancer growth and enhances the efficacy of radiotherapy. Canagliflozin inhibits the cell cycle, mitochondrial respiration, and growth‐promoting pathways. Here, the authors uncovered that canagliflozin mediates its effects partly by suppressing hypoxia‐inducible factor‐1α and histone deacetylase 2.

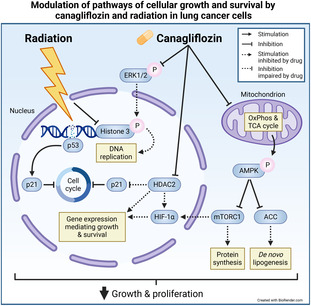

**Abbreviations**
4EBP1: 4E‐binding protein 1
ACC: acetyl‐CoA carboxylase
Akt: protein kinase B
ALDOA: aldolase A
AMPK: adenosine monophosphate‐activated protein kinase
ATCC: American Type Culture Collection
CC3: cleaved caspase 3
*C*_max_
: maximum concentration
DEG: differentially expressed gene
DMEM: Dulbecco's modified eagle's medium
ECAR: extracellular acidification rate
ERK: extracellular signal‐regulated kinase
FDR: false discovery rate
FFPE: formalin‐fixed and paraffin‐embedded
GRA: Glycolytic Rate Assay
H3: histone 3
HDAC: histone deacetylase
HIF‐1α: hypoxia‐inducible factor‐1α
HSA: Highest Single Agent
IC_50_
: half inhibitory concentration
IHC: immunohistochemistry
LA: locally advanced
LDHA: lactate dehydrogenase A
LKB1: liver kinase B1
MAPK: mitogen‐activated protein kinase
MST: Mito Stress Test
mTORC1: mammalian target of rapamycin complex 1
ND1: NADH dehydrogenase 1
NRG: nonobese diabetic rag gamma
NSCLC: non‐small cell lung cancer
OCR: oxygen consumption rate
OxPhos: oxidative phosphorylation
p70S6K: ribosomal protein S6 kinase
PER: proton efflux rate
PFKFB3: 6‐phosphofructo‐2‐kinase/fructose‐2,6‐bisphosphatase 3
PGK1: phosphoglycerate kinase 1
PI: propidium iodide
PI3K: phosphoinositide‐3‐kinase
RNA‐seq: ribonucleic acid sequencing
RPMI: Roswell Park Memorial Institute medium
RT: radiotherapy
RT‐qPCR: real‐time quantitative polymerase chain reaction
S6: ribosomal protein S6
SDS/PAGE: sodium dodecyl‐sulfate polyacrylamide gel electrophoresis
SGLT2: sodium‐glucose cotransporter 2
siRNA: small interfering ribonucleic acid
TCA: tricarboxylic acid

## 1 Introduction

Lung cancer is the leading cause of cancer‐related mortality [1]. At early stages, non‐small cell lung cancer (NSCLC), which represents 85% of lung cancer cases, is reasonably well‐managed with surgery or high‐precision stereotactic radiotherapy (RT) [2]. However, many NSCLC patients present with locally advanced (LA) or metastatic disease at initial diagnosis [3]. Chemotherapy and RT are conventional treatment options for advanced disease, and concurrent chemo‐RT is the therapy of choice for good performance status patients. Nevertheless, response to these therapies is limited as patients with LA‐NSCLC exhibit 5‐year survival rates of 24–32% after concurrent chemo‐RT [4] and metastatic disease is incurable.

In recent years, studies with immune checkpoint inhibitor therapy in addition to chemo‐RT in patients with unresectable LA‐NSCLC improved standard therapy outcomes [5]. However, this treatment also provided moderate improvement in 5‐year survival (42.9% vs 33.4%) and a progression‐free survival rate of 33.1% in patients who showed no progression after chemo‐RT [5]. This underscores an urgent need to explore alternative strategies to augment the efficacy of current NSCLC therapies.

Metabolic reprogramming is a hallmark of cancer, in which cancer cells exhibit distinct metabolic phenotypes to sustain high proliferation rates, adapt to changes in the tumor microenvironment and resist anticancer therapies [6]. The evolutionarily conserved serine/threonine kinase, adenosine monophosphate‐activated protein kinase (AMPK), is a heterotrimeric enzyme consisting of α‐catalytic, and β‐ and γ‐regulatory subunits, which senses metabolic stress and facilitates metabolic reprogramming by orchestrating anabolic and catabolic cellular functions [7]. Elevated AMP/ADP levels bind to AMPK's γ subunit inducing a conformational change and activation of its kinase activity [8]. AMPK inhibits energy expenditure, in part, by suppressing *de novo* lipogenesis, protein synthesis, DNA replication, and the cell cycle; effects mediated through blockade of rate‐limiting enzymes such as acetyl‐CoA carboxylase (ACC), the mammalian target of rapamycin complex 1 (mTORC1) and induction of p53, respectively [7].

Moreover, aberrant activation of the phosphoinositide‐3‐kinase (PI3K)‐protein kinase B (Akt)‐mTORC1 signaling pathway is frequently observed in NSCLC secondary to activating mutations in the epidermal growth factor receptor, Kristen rat sarcoma viral oncogene homolog, or PI3K subunits [9, 10]. Hypoxia‐inducible factor (HIF)‐1α, a regulator of the hypoxic transcriptional response, is stabilized through mTORC1 signaling even under normoxic conditions [11], which contributes to a Warburg glycolytic phenotype [9], radioresistance, and poor prognosis [12, 13]. Hypoxia‐inducible factor‐1α (HIF‐1α) is often overexpressed in NSCLC, and its silencing has been shown to suppress glycolysis and inhibit tumor growth [14, 15, 16]. Thus, the unique ability of the AMPK‐mTORC1 pathway to regulate cancer cell proliferation and survival has triggered interest in exploiting this pathway for cancer therapy.

Canagliflozin, a well‐tolerated oral medication approved for treating type 2 diabetes, was developed to improve glycemia by blocking the renal sodium‐glucose cotransporter 2 (SGLT2) [17]. However, canagliflozin also suppresses mitochondrial complex‐I‐supported respiration, leading to AMPK activation and suppression of the mTORC1 pathway [18]. Our group was the first to demonstrate canagliflozin's antitumor activity in NSCLC and prostate cancer models [19]. Since then, several other groups have shown similar antitumor effects in liver, breast, pancreatic, and other cancers [20]. While some groups have suggested that canagliflozin's anticancer properties are linked to the inhibition of glucose transport via SGLT2 [21, 22, 23], this transporter is functionally present only in certain cancers [23], and emerging evidence indicates that additional mechanisms are at play [24, 25].

Although our earlier work suggested canagliflozin's promising antitumor activity in cultured NSCLC cells, the efficacy of this agent to improve NSCLC response to cytotoxic therapy remains unknown. In this study, we sought to investigate canagliflozin's regulation of the RT and chemotherapy response in cultured cells and human xenograft models and to identify potential mechanisms of action. We report that, within its therapeutic window, canagliflozin inhibits proliferation and tumorigenic potential and enhances cytotoxic therapy response in NSCLC. Furthermore, we show that canagliflozin exerts profound transcriptional reprogramming of key metabolic, growth, and survival pathways, and it exerts its anticancer effects, at least in part, via blockade of histone deacetylase 2 (HDAC2) and the mTORC1‐HIF‐1α signaling pathway.

## 2 Materials and methods

### 2.1 Cell lines and treatments

Human lung A549 (RRID:CVCL_0023), SK‐MES‐1 (RRID:CVCL_0630), H1299 (RRID:CVCL_0060), H1975 (RRID:CVCL_1511), and H460 (RRID:CVCL_0459) cancer cells were obtained from the American Type Culture Collection (ATCC: Manassa, VA). Cell lines were authenticated within the past 3 years using short tandem repeat DNA profiling and comparing the DNA sequences to the reference cell database, with an > 80% match being acceptable. Cells were assessed for mycoplasma contamination using the MycoAlert Mycoplasma Detection Kit (Lonza Group AG: Basel, Switzerland). Cell lines were cultured in DMEM (A549 and SK‐MES‐1), RPMI‐1640 (H1299 and H460) or ATCC‐modified RPMI‐1640 (H1975) with 10% FBS and 1% antibiotic‐antimycotic. All cells were maintained at 37 °C in a humidified 5% CO_2_ incubator. Cells were treated with canagliflozin (MedChemExpress LLC: Monmouth Junction, NJ) solubilized in dimethyl sulfoxide, cisplatin (Accord Healthcare Inc: Durham, NC), etoposide (Teva Canada Ltd: Toronto, Canada), radiation (6MV X‐rays as described [26]), Roxadustat (FG‐4592), BAY‐87‐2243, and IACS‐010759 (Cayman Chemical Company: Ann Arbor, MI).

### 2.2 Proliferation

Cells (500 cells/well) were seeded in 96‐well plates and left to adhere overnight. When untreated control wells reached 80% confluency, cells were fixed with 10% formalin and stained with 0.5% crystal violet. The stain was solubilized with 20% MeOH, and absorbance was measured at 570 nm on the SpectraMax iD5 Multi‐Mode Microplate Reader (Molecular Devices: San Jose, CA).

### 2.3 Clonogenic survival

Cells were seeded in 12‐well plates at different densities depending on the RT dose (500 cells/well for 0 Gy, 1 × 10^3^ cells/well for 2 Gy and 2 × 10^3^ cells/well for 4 Gy) and left to adhere overnight. After a 7‐day incubation post‐treatment administration or when viable colonies (defined as a cluster of ≥ 50 cells) formed in the untreated control wells, cells were fixed with 10% formalin, stained with crystal violet and colonies were counted. Data were normalized to seeding density.

### 2.4 Synergy analysis

The Highest Single Agent (HSA) model on the SynergyFinder web application (https://synergyfinder.org) was used to predict additivity, synergism, or antagonism of combined treatments [27].

### 2.5 Xenograft models

**Fig. 1:**
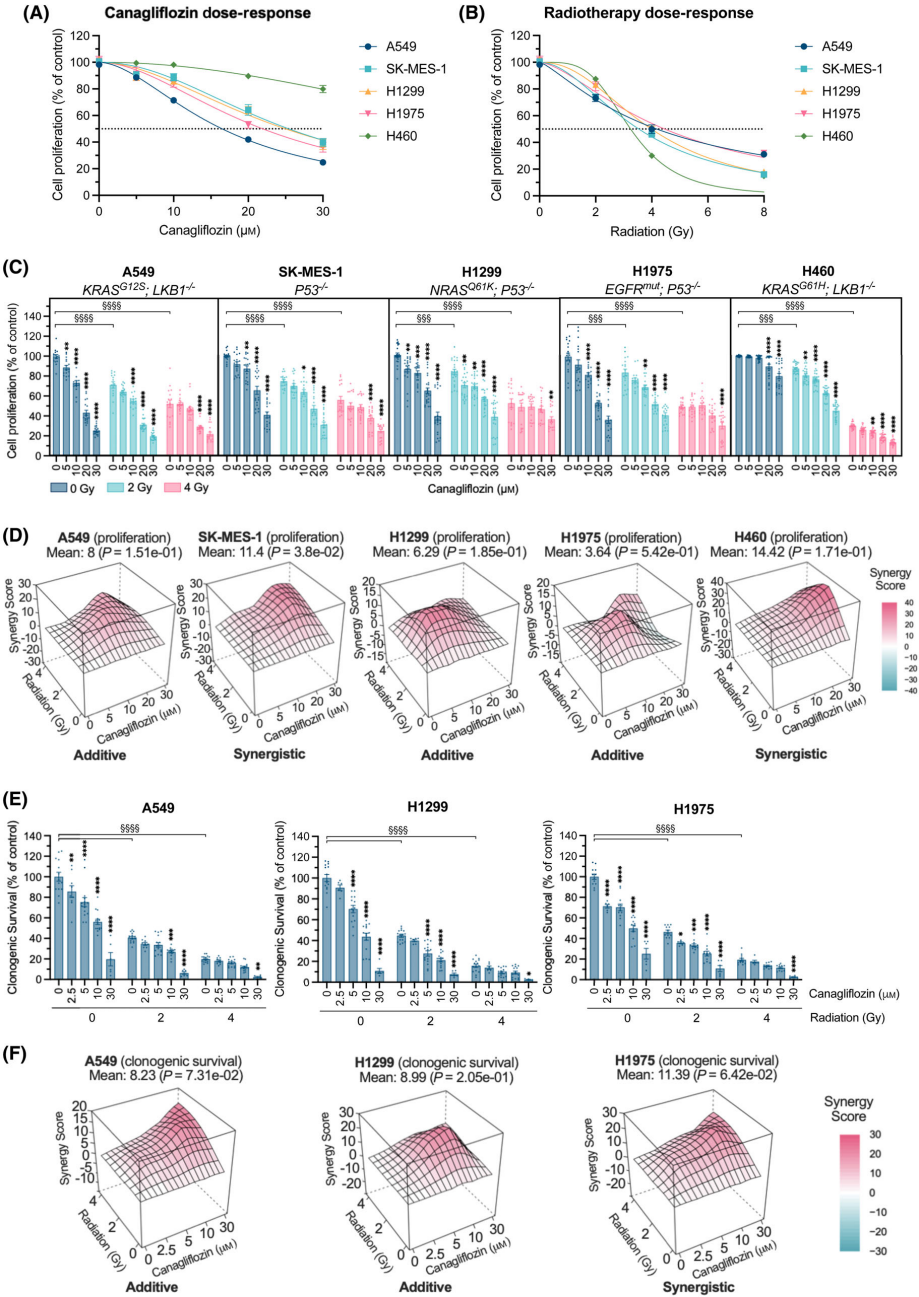
Anticancer efficacy of NSCLC cells treated with canagliflozin and RT. Proliferation assay with human NSCLC cells (A549, SK‐MES‐1, H1299, H1975, and H460) treated with (A) canagliflozin [5–30 μm; half inhibitory concentration (IC_50_) values: 16.4, 25.4, 25.0, 21.9, and > 30 μm, respectively], (B) RT (single fraction of 2–8 Gy; IC_50_ values: 4.2, 3.5, 4.0, 4.5, and 3.2 Gy, respectively) or (C) a combination treatment (5–30 μm canagliflozin and/or 2 or 4 Gy RT). The dotted line indicates a 50% reduction in cell proliferation. Cells were pretreated with canagliflozin for 5 h before RT and incubated until untreated control wells reached ~ 80% confluency (5 days). (D) Proliferation assay results were subjected to synergy analysis using HSA modeling where a mean HSA synergy score of < −10 suggests antagonism, −10–10 suggests additivity, and > 10 suggests synergy. (E) Clonogenic survival assay with human NSCLC cells (A549, H1299, and H1975) treated with canagliflozin (2.5–30 μm) and/or RT (single fraction of 2 or 4 Gy) treatments. Cells were pretreated with canagliflozin 5 h prior to RT and incubated until viable colonies (cluster of ≥ 50 cells) formed in the untreated control wells (7 days). (F) Clonogenic survival data were analyzed using HSA synergy modeling. Results were derived from three independent experiments. All data are presented as mean ± SEM and calculated using two‐way ANOVA (C, E) followed by Tukey's multiple comparisons test. *P < 0.05, **P < 0.01, ***/^§§§^ *P* < 0.001, ****/^§§§§^ *P* < 0.0001.

Six‐ to eight‐week‐old male athymic BALB/c nude mice (CAnN.Cg‐Foxnnu/Crl; Charles River: Wilmington, MA) and male and female nonobese diabetic rag gamma (NRG) mice (NOD.Cg‐Rag1(tm1Mom)Il2rg(tm1Wjl)/SzJ; bred in‐house; Jackson Laboratory: Bar Harbor, ME) were housed in a pathogen‐free facility with *ad libitum* access to food and water. NSCLC cells (1 × 10^6^ cells) were injected subcutaneously into flanks of anesthetized mice. Tumors were measured with a caliper, and volume was calculated using V = ½ × (length×width^2^). Animals were treated as described in Fig. 1 legend. Tumors were formalin‐fixed and paraffin‐embedded (FFPE) by the McMaster Histology Core Facility. The McMaster University Animal Ethics Research Board approved all animal procedures (animal utilization protocol 20‐12‐47).

### 2.6 Canagliflozin diet

Envigo (Indianapolis, IN) prepared the diet by supplementing standard chow diet (#2018) with powdered 300 mg Invokana tablets (83.3% purity; Janssen Pharmaceuticals: Beerse, Belgium) to a final concentration of 416.7 ppm (347.24 mg·kg^−1^; daily dose of 60 mg·kg^−1^). Values were based on mouse body weights during the experimental course (22–30 g) and daily diet intake of 2.5–6 g·day^−1^ (approximately 60% intake and 40% waste).

### 2.7 Functional metabolism assays

Cells were seeded at 2 × 10^4^ cells/well, treated as indicated, and analyzed on the Seahorse Bioscience XFe96 Extracellular Flux Analyzer (Agilent Technologies: Santa Clara, CA). After a 48‐h incubation with the treatments, cells were subjected to the Glycolytic Rate Assay (GRA) or the Mito Stress Test (MST) as per the manufacturer's protocol [28]. Cells were assayed in Seahorse XF DMEM (25 mm d‐glucose, 1 mm sodium pyruvate, and 4 mm l‐glutamine) without drug treatments. GRA inhibitors: 0.5 μm rotenone/antimycin A and 50 mm 2‐DG. MST inhibitors: 1.5 μm oligomycin, 1 μm FCCP, and 0.5 μm rotenone/antimycin A. After the assays, cells were stained with crystal violet as described in Section 2.2, and data were normalized to cell content. Wave Desktop software was used for data acquisition and analysis (version 2.6; Agilent Technologies).

### 2.8 Immunoblotting

Cells were lysed as described [19], and protein concentrations were determined using the BCA Protein Assay Kit (Thermo Fisher Scientific: Waltham, MA). Lysates were subjected to electrophoresis (SDS/PAGE), immunoblotting [19], and imaging on a Fusion FX system (Vilber Lourmat: Collégien, France). See Table S1 for list of antibodies.

### 2.9 Immunohistochemistry (IHC)

Standard IHC protocols were followed on FFPE tumor sections as described [29]. Tissues were incubated overnight with the indicated primary antibody at 4 °C, followed by biotinylated secondary antibody and HRP streptavidin. Slides were developed using the Vector NovaRED Substrate Kit (Vector Laboratories Inc: Burlingame, CA). Hematoxylin was used as a counterstain. See Table S1 for list of antibodies.

### 2.10 IHC quantification and necrosis evaluation

For evaluation of p‐ACC^Ser79^, CC3^Asp175^, and p‐H3^Ser10^, 10 random high‐power fields (40×) of each slide were quantified. The cytoplasmic intensity for p‐ACC^Ser79^ and the percentage of nuclear positivity for CC3^Asp175^ and p‐H3^Ser10^ were quantified using imagej software (version 1.53a; National Institutes of Health: Bethesda, MD). For necrosis evaluation, the ratio of necrotic area to whole tumoral section was quantified using imagej [30].

### 2.11 Cell cycle analysis

Following treatment, cells were fixed in 70% ethanol and stained with FxCycle Propidium Iodide (PI)/RNase Staining Solution (Thermo Fisher Scientific). At least 1 × 10^5^ events/sample were analyzed on the CytoFLEX LX Flow Cytometer (Beckman Coulter: Brea, CA; 488 nm excitation and 610 nm emission). flowjo software (version 10.8.0; FlowJo LLC: Ashland, OR) was used for analysis.

### 2.12 Real‐time quantitative PCR (RT‐qPCR)

The RNeasy Mini Kit (QIAGEN: Hilden, Germany) was used for total RNA purification. Samples were reverse transcribed to cDNA using SuperScript IV reverse transcriptase (Invitrogen: Waltham, MA). Amplification and detection were performed in a qPCR thermocycler (Corbett Rotor Gene 6000: Montreal Biotech Inc: Dorval, Canada) using TaqMan Assay fluorogenic 5′ nuclease chemistry (Invitrogen). Relative gene expression was calculated using the Livak method [31] normalizing to GAPDH. See Table S1 for list of probes.

### 2.13 RNA sequencing (RNA‐seq)

Purified RNA samples were subjected to RNA‐seq at the McMaster Genomics Facility. RNA quality was confirmed using the 2100 Bioanalyzer System (Agilent Technologies). mRNA was enriched from 1 μg of total RNA using the NEBNext Poly(A) mRNA Magnetic Isolation Module, and Illumina libraries were prepared using the NEBNext Ultra II Directional RNA Library Prep Kit (New England Biolabs: Ipswich, MA). Each run was done with the Illumina HiSeq 1500 using the HiSeq Rapid v2 chemistry with onboard cluster generation and a 1 × 50 bp configuration (Illumina: San Diego, CA), aiming for approximately 25 M clusters per sample.

### 2.14 Differential gene expression analysis

Sequencing data were uploaded to the Galaxy web platform, and the public server at usegalaxy.org was used for data processing [32]. Briefly, raw reads underwent quality control and alignment against the GRCh38/hg38 genome using FastQC (http://www.bioinformatics.babraham.ac.uk/projects/fastqc/), Cutadapt [33] and HISAT2 [34]. Aligned reads per gene were then processed through featureCounts and DESeq2 [35] to identify differentially expressed genes (DEGs) with an adjusted *P‐*value (*q*‐value) < 0.05.

### 2.15 Gene set and regulatory network analysis

Gene ontology analysis was performed using Gene Set Enrichment Analysis (version 4.1.0) [36]. Cytoscape's iRegulon plugin (version 3.9.1) was used to predict the regulators responsible for modulating gene expression.

### 2.16 siRNA transcript targeting

Cells were transfected with HIF‐1α FlexiTube small interfering ribonucleic acid (siRNA; Hs_HIF1A_6, SI02664431; QIAGEN), HDAC2 FlexiTube siRNA (Hs_HDAC2_4, SI00434973; QIAGEN), and the HiPerFect Transfection Reagent (QIAGEN) as per the manufacturer's protocol. The AllStars Negative Control siRNA (QIAGEN) was used as the negative control.

### 2.17 Prognostic analysis

The Kaplan–Meier plotter (https://kmplot.com/analysis/) was used to analyze gene expression and survival data of 1925 lung cancer cases from 12 cohorts [37].

### 2.18 Statistical analyses

Ordinary one‐way or two‐way ANOVA followed by Tukey's *post hoc* test was used to evaluate the significance between treatments unless otherwise indicated. Proliferation and clonogenic survival IC_50_ values were calculated using a nonlinear regression model with a normalized slope. Statistical analyses were performed using graphpad prism (version 9.1.0; GraphPad: San Diego, CA). Statistical significance was considered at *P*‐value < 0.05.

## 3 Results

### 3.1 Antitumor efficacy

#### 3.1.1 Proliferation

We examined the antiproliferative effects of clinically relevant doses of canagliflozin (5–30 μm) and RT; 2 or 4 Gy in human adenocarcinoma (A549, H1299 and H1975), squamous cell (SK‐MES‐1), and large cell (H460) NSCLC cells. Canagliflozin and RT dose‐dependently inhibited the proliferation of all cell lines, with A549 cells showing the highest sensitivity to canagliflozin and H460 the least (Fig. 1A,B; see figure legend for IC_50_ values). All NSCLC cell lines treated with RT exhibited additional reduction of proliferation with canagliflozin (Fig. 1C). Synergy analysis revealed that the addition of canagliflozin to RT provided additive antiproliferative effects in A549, H1299, and H1975 cells and synergistic effects in SK‐MES‐1 and H460 cells (Fig. 1D).

#### 3.1.2 Clonogenic survival

In clonogenic survival assays, canagliflozin (2.5–30 μm) suppressed the clonogenic potential of A549, H1299, and H1975 cells (IC_50_ values: 4.4, 5.7, and 7.2 μm, respectively) alone and in combination with RT (2 or 4 Gy; Fig. 1E). Suppression of survival by canagliflozin and RT was additive in A549 and H1299 cells and synergistic in H1975 cells (Fig. 1F). These data demonstrate that clinically achievable concentrations of canagliflozin effectively inhibit tumorigenic potential and enhance the efficacy of RT in NSCLC cells.

#### 3.1.3 Tumor growth

**Fig. 2:**
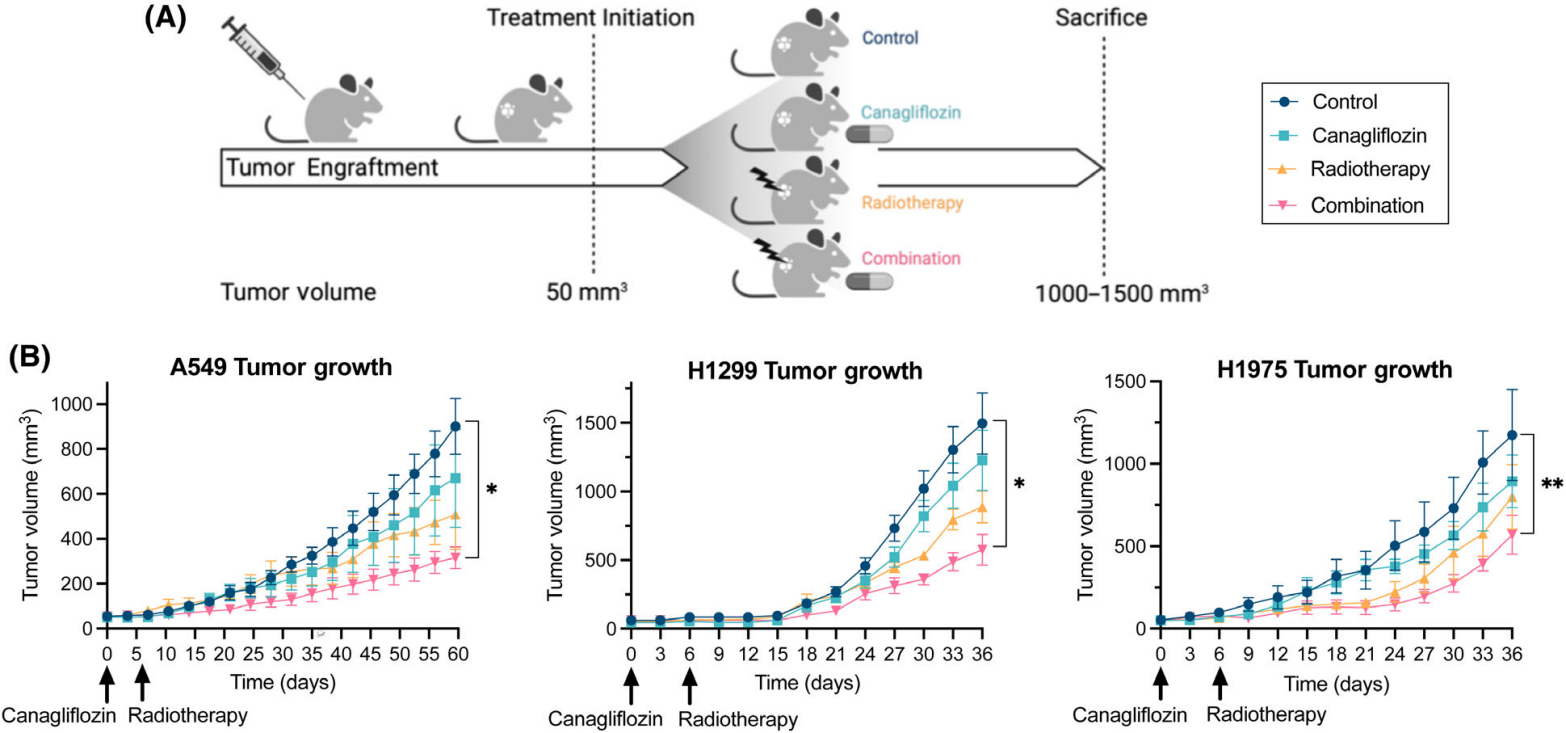
Antitumor efficacy of NSCLC xenografts treated with canagliflozin and RT. (A) A schematic of the treatment regimen for ectopic xenografts (created with BioRender.com). When tumors reached 50 mm^3^, mice were randomly assigned to one of four treatment groups: (1) control, (2) canagliflozin (delivered through diet; see Section 2), (3) RT (single fraction of 5 Gy), or (4) combination treatment. Mice receiving RT were treated 6‐day post‐treatment initiation with 6MV parallel opposed X‐ray beams. (B) Tumor growth kinetics of A549 (grown in BALB/c nude mice; *n* = 4–5/group; 16 animals total), H1299 (grown in NRG mice; *n* = 5/group; 20 animals total) and H1975 (grown in NRG mice; *n* = 5/group; 20 animals total) xenografts treated with vehicle control, canagliflozin, RT, or combination therapy. Mice were sacrificed when tumors reached 1000–1500 mm^3^. All data are presented as mean ± SEM and calculated using repeated measures ANOVA (B) followed by Tukey's multiple comparisons test. *P < 0.05, **P < 0.01.

We investigated the effects of oral canagliflozin (diet designed to deliver 60 mg·kg^−1^·day^−1^; see Section 2), RT (5 Gy) and combination therapy in A549, H1299, and H1975 xenograft models (Fig. 2A). Consistent with previous studies in rodents and humans [38], the polyuria induced by canagliflozin led to greater food and water intake and resulted in lower body mass compared with mice fed the control diet (Fig. S1A–E). The estimated daily dose of canagliflozin in mice harboring A549, H1299, and H1975 tumors was 81, 55, and 78 mg·kg^−1^·day^−1^, respectively.

Canagliflozin and RT monotherapies reduced tumor growth by 18–26% and 32–44% compared with control mice; however, this did not reach statistical significance at endpoint (Fig. 2B). In contrast, the combination therapy significantly suppressed tumor growth in all three cohorts (51–65% tumor suppression compared with controls).

### 3.2 Mechanism of action

#### 3.2.1 Regulation of gene expression: Induction of oxidative phosphorylation (OxPhos) and downregulation of hypoxia pathways

**Fig. 3:**
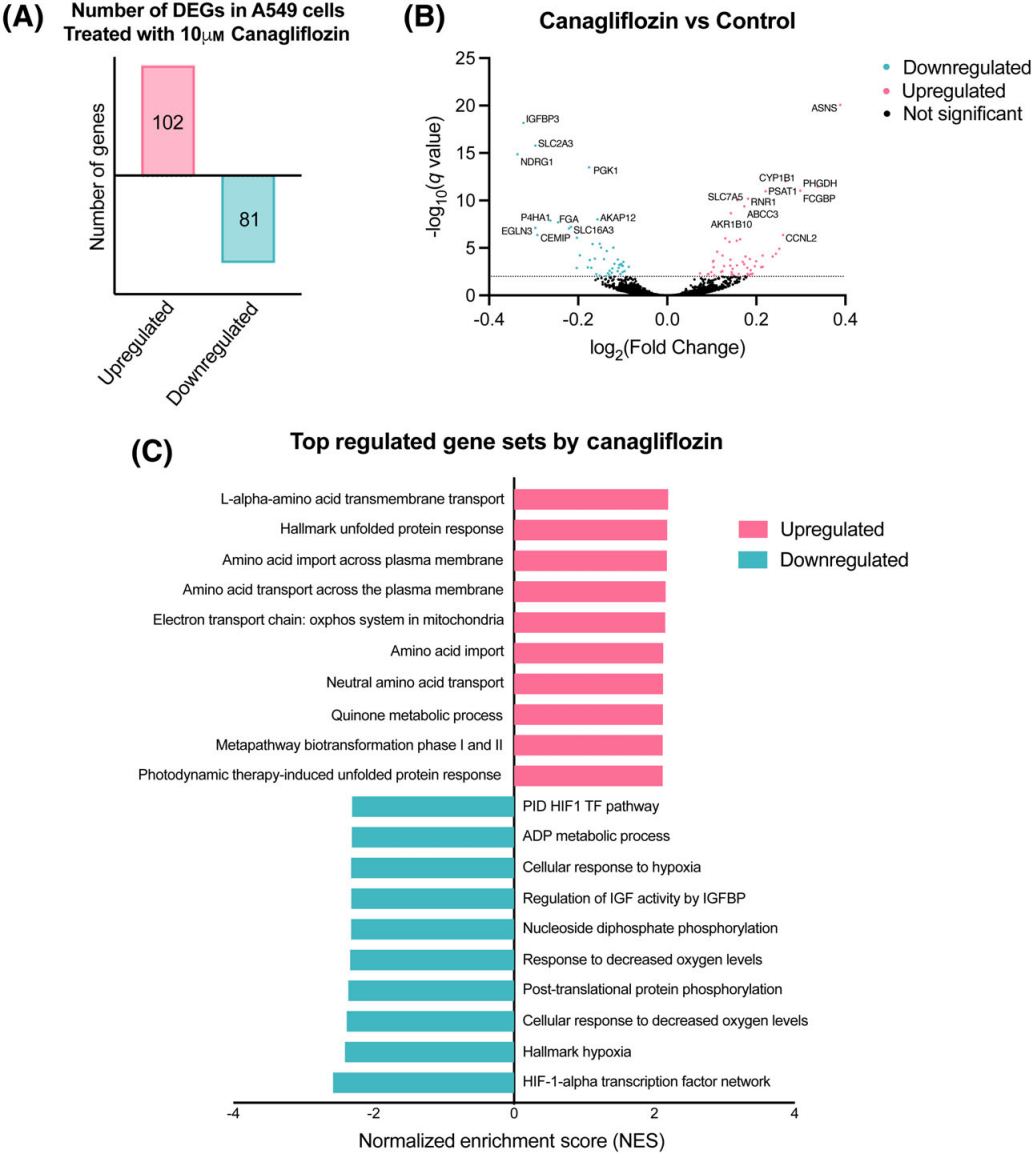
Bioinformatics analysis of DEGs in A549 NSCLC cells treated with canagliflozin. RNA‐seq was employed to assess the transcriptome profile of A549 cells treated with 10 μm canagliflozin for 24 h (*n* = 3). (A) The number of significantly upregulated and downregulated DEGs in A549 cells following canagliflozin treatment with a FDR *q*‐value < 0.05. (B) A volcano plot depicting the total upregulated (positive log_2_(Fold Change)) and downregulated (negative log_2_(Fold Change)) genes by canagliflozin. DEGs with a −log_10_(*P*‐value) < 2 are separated with a dotted line and have an FDR *q*‐value < 0.05. (C) The most significantly regulated gene sets from Gene Set Enrichment Analysis of canagliflozin‐treated A549 cells. Data were derived from a single RNA‐seq experiment.

To identify potential mechanisms contributing to these effects of canagliflozin and RT, we conducted RNA‐seq analysis and found that canagliflozin (10 μm) significantly upregulated 102 and downregulated 81 genes (Fig. 3A), whereas RT (5 Gy) upregulated 2562 and downregulated 3226 genes (Fig. S2A). The volcano plot in Fig. 3B illustrates the most significantly downregulated genes by canagliflozin (*IGFBP1*, *SLC2A3*, *NDRG1*, *PGK*1, *EGLN3*, and *P4HA1*; involved in growth factor activity, glucose uptake and glycolysis, cell growth and differentiation, and response to hypoxia) and upregulated genes (*ASNS*, *PHGDH*, *PSAT1*, *SLC1A5*, *CYP1B1*, and *RNR1*; and involved in amino acid uptake, metabolism, and ribosomal function). To understand the biological processes behind the DEGs, DEGs were subjected to Gene Set Enrichment Analysis. The majority of significantly downregulated pathways were associated with cellular response to hypoxia, whereas the most significantly upregulated pathways include amino acid transport, unfolded protein response, and mitochondrial OxPhos (Fig. 3C). Interestingly, a large proportion of genes modulated by canagliflozin (146 of 183 DEGs) were also influenced by RT (Fig. S2B).

#### 3.2.2 Mitochondrial respiration

**Fig. 4:**
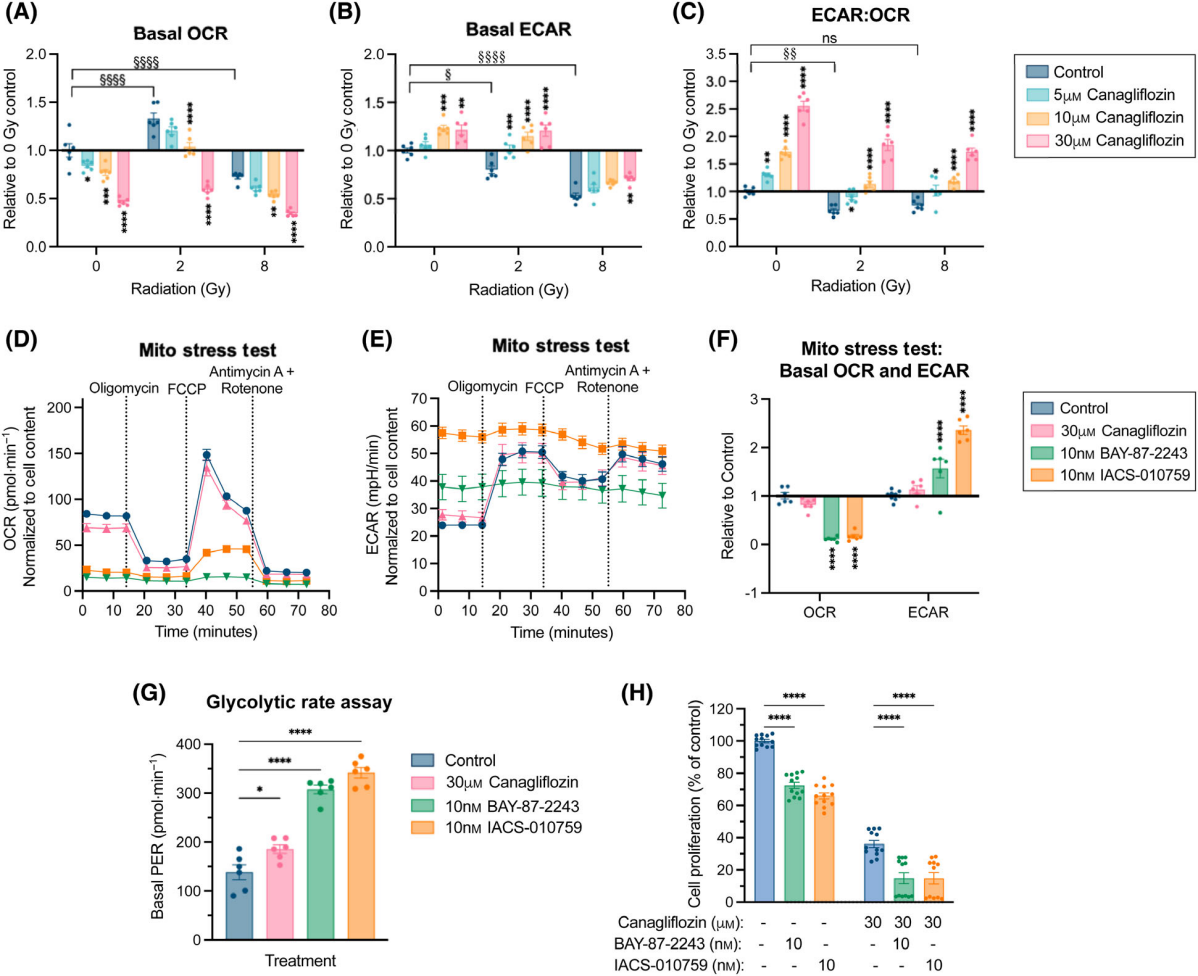
Effects of canagliflozin and RT on mitochondrial respiration in A549 NSCLC cells. A549 cells were pretreated for 5 h with the indicated canagliflozin (5–30 μm) doses prior to RT (single fraction of 2 or 8 Gy), and OCR and ECAR were measured with the Seahorse MST 48 h after RT. Effects of the treatments on (A) basal OCR, (B) basal ECAR, and (C) basal ECAR/OCR ratio were plotted relative to the untreated control. See Fig. S3 for time‐dependent data from the MST. A549 cells were treated with canagliflozin (30 μm) and the mitochondrial complex‐I inhibitors, BAY‐87‐2243 (10 nm) or IACS‐010759 (10 nm), for 48 h and subjected to the Seahorse MST to measure (D) OCR and (E) ECAR. (F) Basal OCR and ECAR values from ‘D’ and ‘E’ were plotted relative to the untreated control. (G) A549 cells were treated for 48 h with canagliflozin (30 μm), BAY‐87‐2243 (10 nm), or IACS‐010759 (10 nm) and subjected to the Seahorse GRA to measure the basal PER. All Seahorse data were normalized to cell content. (H) A proliferation assay of A549 cells treated with BAY‐87‐2243 (10 nm), IACS‐010759 (10 nm), and/or canagliflozin (30 μm) for 5 days. Drug treatments were administered to the cells concurrently. All experiments were obtained from three independent experiments and presented as mean ± SEM. Data were analyzed using two‐way ANOVA (A–C, F, G) or ordinary one‐way ANOVA (H) followed by Tukey's multiple comparisons test. ns: nonsignificant, */^§^ *P* < 0.05, **/^§§^ *P* < 0.01, ***P < 0.001, ****/^§§§§^ *P* < 0.0001.

Given the observed modulation of OxPhos gene expression, we examined cellular respiration. In nonirradiated cells, canagliflozin (5–30 μm) caused small but significant dose‐dependent suppression of the oxygen consumption rate (OCR) and enhancement of the extracellular acidification rate (ECAR; Fig. 4A–C, Fig. S3A,B). RT (2 and 8 Gy) did not alter OCR consistently but significantly repressed ECAR (Fig. 4A,B). The addition of canagliflozin to RT suppressed OCR and reversed the RT‐induced inhibition of ECAR (Fig. 4A,B). The ECAR/OCR ratio, an indicator of anaerobic metabolism (metabolic stress), was reduced with RT but was effectively enhanced by canagliflozin in both nonirradiated and irradiated cells (Fig. 4C). Overall, canagliflozin mediated modest metabolic stress in NSCLC cells as indicated by changes of 54% or lower in OCR or ECAR.

To better understand canagliflozin's impact on mitochondrial respiration, we compared its effects to those of the potent and specific OxPhos complex‐I inhibitors, BAY‐87‐2243 and IACS‐010759. Seahorse MST and GRAs showed that a widely used dose (10 nm) of BAY‐87‐2243 or IACS‐010759 dramatically blocked OCR (91–94% inhibition) and induced ECAR (1.6–2.4‐fold increase) compared with untreated cells (Fig. 4D–F). The evaluated proton efflux rate (PER) increased by 2.2–2.5‐fold with BAY‐87‐2243 and IACS‐010759, respectively, compared with a minor increase (1.3‐fold) by canagliflozin (Fig. 4G), indicating a greater induction of acidification of the extracellular space. Interestingly, BAY‐87‐2243 and IACS‐010759 induced lower suppression of proliferation (28–34%) in A549 cells compared with that achieved by 30 μm canagliflozin (64%) and the addition of canagliflozin to those agents provided increased antiproliferative capacity (Fig. 4H).

#### 3.2.3 Regulation of metabolic stress and growth factor pathways

**Fig. 5:**
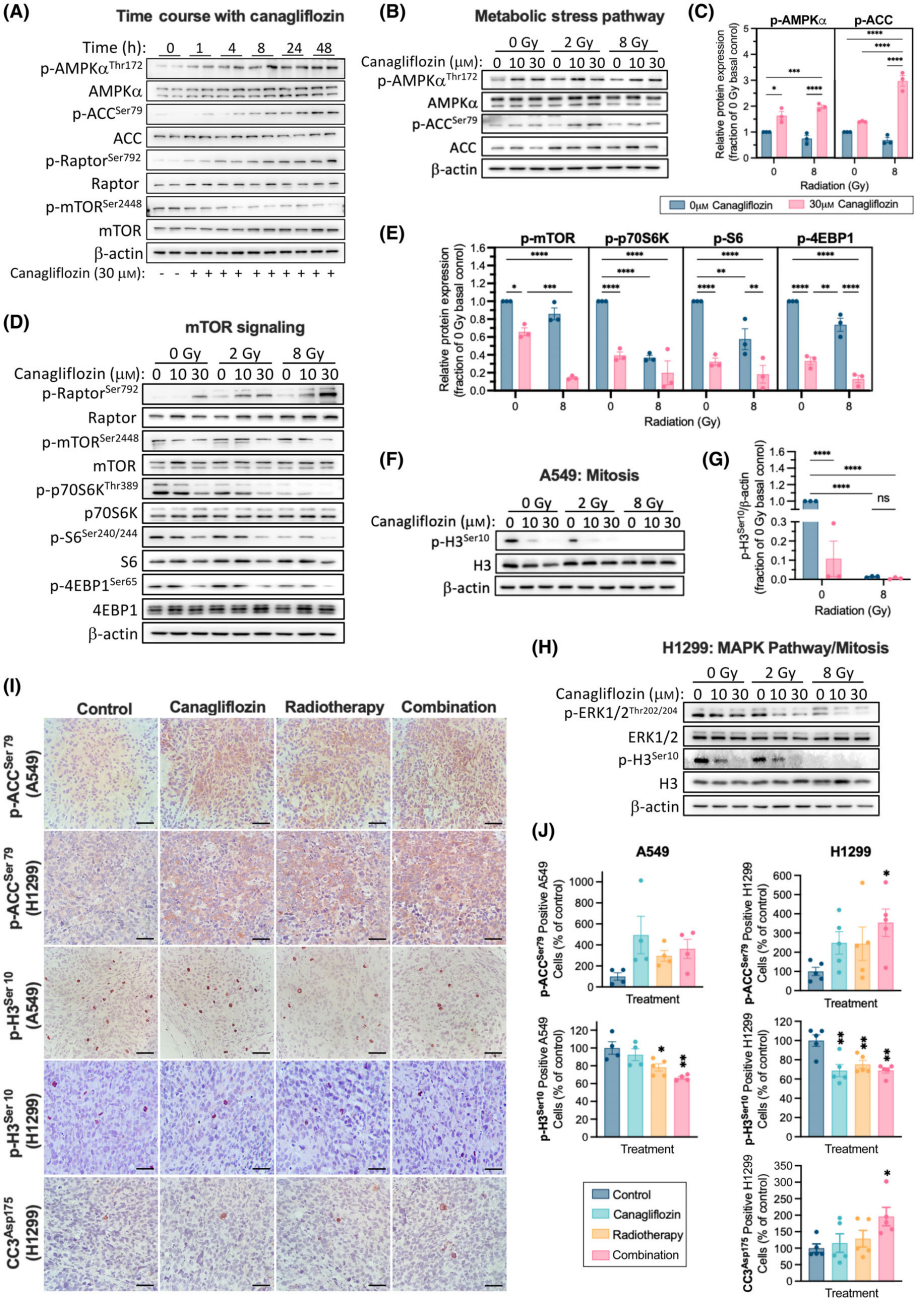
Regulation of metabolic and cell proliferation pathways by canagliflozin and RT in NSCLC. (A) Representative protein immunoblots of metabolic stress markers in A549 cells treated with canagliflozin (30 μm) for the indicated duration (1–48 h). Representative protein immunoblots of A549 cells pretreated with canagliflozin (10 or 30 μm) for 5 h and subsequently treated with RT (single fraction of 2 or 8 Gy) and probed for enzymes involved in the (B) metabolic stress pathway (corresponding quantification in (C)), (D) mTOR signaling (quantification in (E)), and (F) mitosis (quantification in (G)) 48 h after RT. (H) Representative immunoblots of H1299 cells pretreated with canagliflozin (10 or 30 μm) for 5 h and subsequently treated with RT (single fraction of 2 or 8 Gy). Forty‐eight hours after RT, lysates were probed for enzymes involved in the MAPK/ERK pathway and mitosis. (I) Representative images of A549 (*n* = 4–5/group) and H1299 (*n* = 5/group) tumor sections analyzed by IHC for phospho‐histone 3 (p‐H3^Ser10^), phospho‐acetyl‐CoA carboxylase (p‐ACC^Ser79^), and cleaved caspase 3 (CC3^Asp175^; scale bar = 100 μm). Tumors were treated as shown in Fig. 2A and harvested for IHC analysis at day 60 or 36 for A549 and H1299 tumors, respectively. IHC quantifications are illustrated in panel (J). Western blot data were obtained from three independent experiments, normalized to β‐actin, and presented as mean ± SEM. Two‐way ANOVA (C, E, G) followed by Tukey's multiple comparisons test or ordinary one‐way ANOVA (J) followed by Bonferroni's multiple comparisons test was used to detect statistical significance. ns: nonsignificant, *P < 0.05, **P < 0.01, ***P < 0.001, ****P < 0.0001.

To understand the time course of metabolic stress signals induced by canagliflozin, we analyzed the effects of canagliflozin (30 μm) in A549 cells at different time points (Fig. 5A). The activating AMPK Thr172 phosphorylation (p‐AMPKα^Thr172^) was detected within 1 h of drug incubation but continued to increase up to 48 h. The inhibitory phosphorylation of Ser79 on ACC (p‐ACC^Ser79^) and Ser792 on Raptor (p‐Raptor^Ser792^), direct AMPK targets [39, 40], was detected by 8 h and maintained for up to 48 h. Consistent with these effects, suppression of Ser2448 phosphorylation on mTOR (p‐mTOR^Ser2448^) continued to 48 h (Fig. 5A). Therefore, subsequent analyses were performed after 48 h of canagliflozin treatment.

To examine the metabolic effects of canagliflozin in combination with RT, cells were pretreated for 5 h with 10 or 30 μm canagliflozin and analyzed 48 h after 2 or 8 Gy RT exposure. Canagliflozin alone induced p‐AMPKα^Thr172^ and p‐ACC^Ser79^ in nonirradiated and irradiated cells indicating activation of metabolic stress signaling (Fig. 5B,C). The mTOR pathway was suppressed by canagliflozin, and RT provided further inhibitory effects (Fig. 5D). This is evident by the inhibition of p‐mTOR^Ser2448^ and its downstream targets: p‐p70S6K^Thr389^, p‐S6^Ser240/244^, and p‐4EBP1^Ser65^ (Fig. 5E). Similar results were obtained in H1299 cells (Fig. S4A,B).

The marked suppression of the above pathways by canagliflozin suggested that the drug may also suppress DNA replication and mitosis. Indeed, phosphorylated histone 3 (Ser10; p‐H3^Ser10^), a marker identifying mitotic cells, was abolished by canagliflozin and RT in both A549 and H1299 cells (Fig. 5F–H). This was associated with suppression of the activating phosphorylation of the p‐H3^Ser10^ upstream kinase, extracellular signal‐regulated kinase (ERK)‐mitogen‐activated protein kinase (MAPK) ERK1/2 (Thr202/204) [41] (Fig. 5H), indicating that canagliflozin effectively suppresses multiple growth factor signaling pathways.

#### 3.2.4 Proliferation, metabolic and apoptotic markers in tumors

We analyzed A549 and H1299 xenograft tumors with immunohistochemistry (IHC) using antibodies specific for p‐H3^Ser10^, p‐ACC^Ser79^, or cleaved caspase 3 (CC3^Asp175^; Fig. 5I). Canagliflozin, RT, and even more so the combined therapy mediated marked induction of p‐ACC^Ser79^ reaching statistical significance in H1299 tumors (*P* = 0.0390; Fig. 5J).

Compared with untreated controls, canagliflozin, RT, and the combined treatment reduced the proportion of p‐H3^Ser10^‐positive cells in A549 and H1299 tumors, with the combination treatment showing more apparent additive effects in A549 tumors (Fig. 5J). Furthermore, IHC for CC3^Asp175^ detected low but increasing numbers of apoptotic cells in tumors treated with canagliflozin, RT, and combination therapy in H1299 tumors (*P* = 0.0401; Fig. 5J). However, we could not detect consistent changes in levels of tumor necrosis in response to any of the treatments (Fig. S5).

#### 3.2.5 Regulation of cell cycle checkpoints

**Fig. 6:**
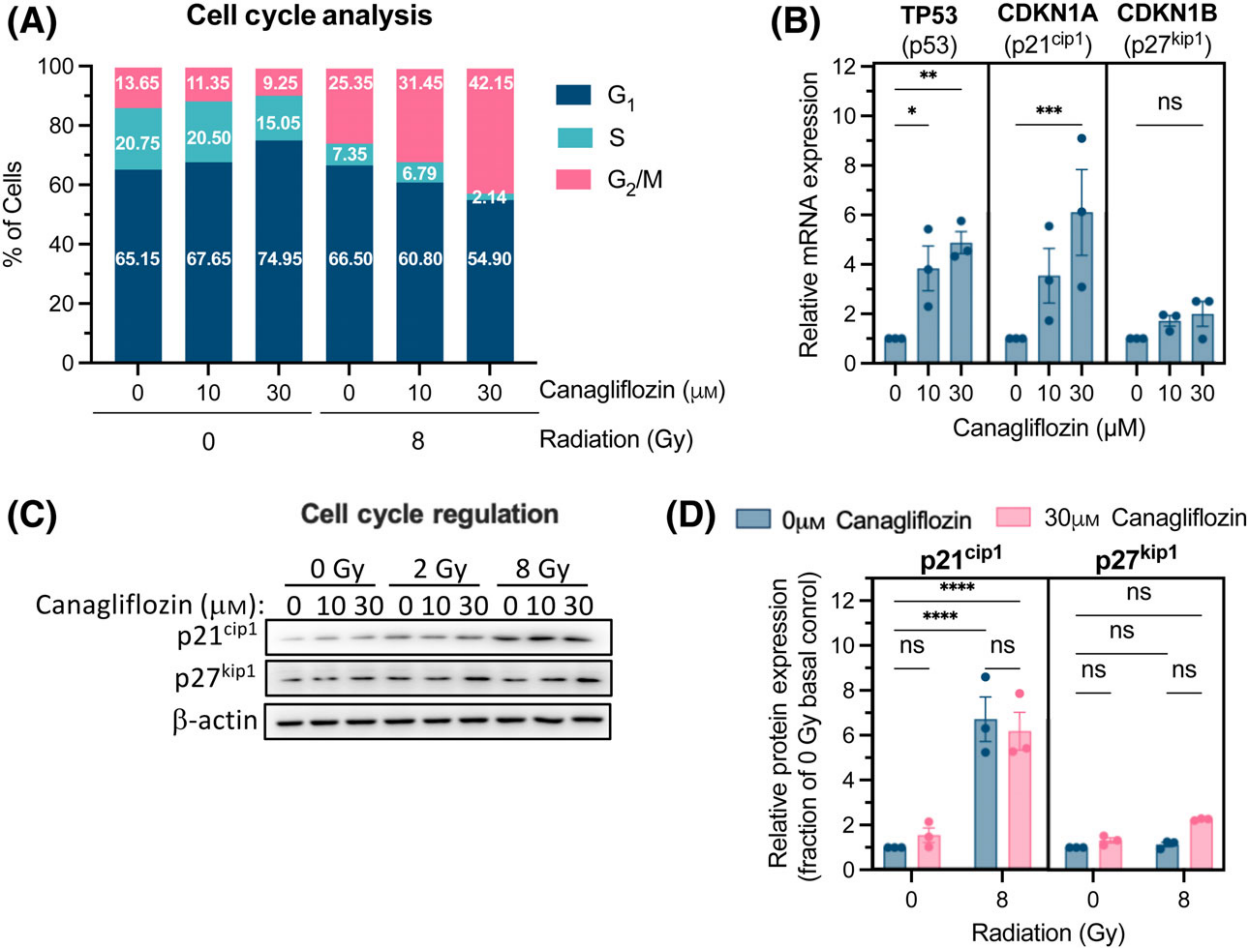
Regulation of the cell cycle by canagliflozin and RT in A549 NSCLC cells. (A) Cell cycle analysis using flow cytometry of A549 cells pretreated for 5 h with canagliflozin (10 or 30 μm) before RT (single fraction of 8 Gy) and analyzed 48 h later. (B) Relative mRNA expression, determined with RT‐qPCR, of cell cycle regulation genes in A549 cells treated with canagliflozin (10 or 30 μm) for 2 h. See Fig. S6 for the canagliflozin time course up to 24 h. (C) Representative protein immunoblots of cell cycle regulation markers, p21^cip1^ and p27^kip1^, in A549 cells pretreated for 5 h with canagliflozin (10 or 30 μm) followed by RT (single fraction of 2 or 8 Gy) and analyzed 48 h later (quantification in (D) normalized to β‐actin). Data were obtained from three independent experiments and presented as mean ± SEM. Two‐way ANOVA (D) or ordinary one‐way ANOVA (B) followed by Tukey's multiple comparisons test was used to detect statistical significance. ns: nonsignificant, *P < 0.05, **P < 0.01, ***P < 0.001, ****P < 0.0001.

Canagliflozin (10 and 30 μm) mediated dose‐dependent accumulation of nonirradiated cells in the G_1_ phase (0 vs 30 μm *P* = 0.0065; Fig. 6A). However, when combined with 8 Gy, there was increased cell distribution in the radiosensitive G_2_/M phase (25.35 vs 42.15%; *P* < 0.0001).

Quantitative real‐time PCR (RT‐qPCR) showed that canagliflozin, in a dose‐dependent fashion, regulated genes involved in G_1_/S and G_2_/M checkpoints, such as *TP53* (p53) and the cyclin‐dependent kinase inhibitors, *CDKN1A* (p21^cip1^) and *CDKN1B* (p27^kip1^; Fig. 6B). Transcript levels rose transiently with canagliflozin treatment at 2 h and returned to lower levels by 24 h (Fig. S6). Consistent with modulation of the cell cycle, canagliflozin alone caused a slight nonsignificant increase in p21^cip1^ and p27^kip1^ protein levels after a 48‐h treatment (Fig. 6C,D). While RT led to significant induction of p21^cip1^ protein levels, we did not detect further significant induction of p21^cip1^ or p27^kip1^ protein beyond that caused by RT (Fig. 6C,D).

#### 3.2.6 HIF‐1α inhibition as a potential mechanism of action

**Fig. 7:**
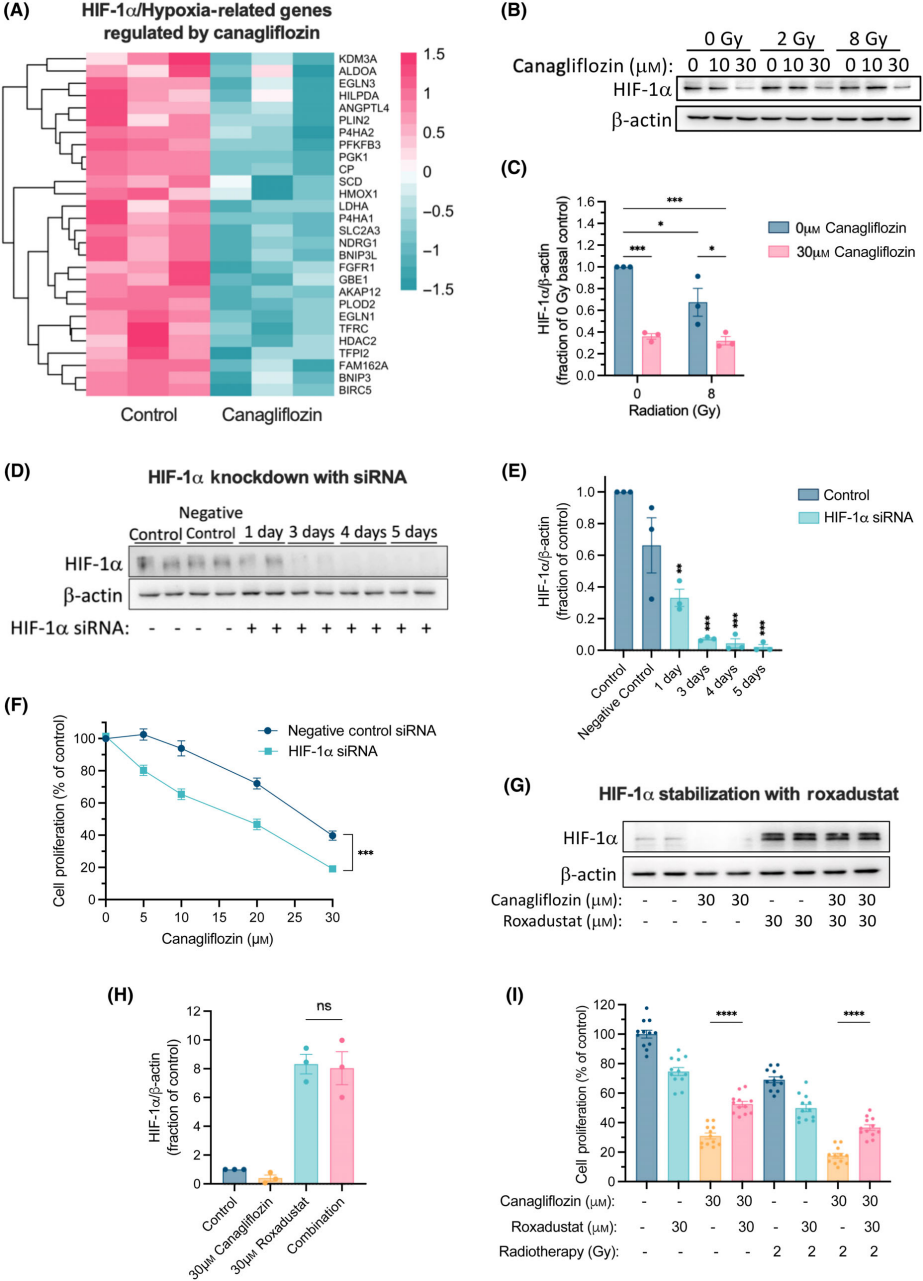
Analysis of HIF‐1α regulation in A549 NSCLC cells treated with canagliflozin. (A) A heatmap illustrating HIF‐1α‐related genes by 10 μm canagliflozin for 24 h in A549 cells (*n* = 3; FDR *q*‐value < 0.05). Data were obtained from the RNA‐seq experiment. The teal‐to‐pink scale represents downregulated to upregulated from −1.5 to 1.5, respectively. (B) Representative protein immunoblots of A549 cells pretreated with canagliflozin (10 or 30 μm) for 5 h and subsequently treated with RT (single fraction of 2 or 8 Gy) and probed for HIF‐1α 48 h later (corresponding quantification in (C)). (D) Representative protein immunoblots and (E) quantification of HIF‐1α in A549 cells treated with negative control siRNA (5 nm) or HIF‐1α siRNA (5 nm) for the indicated duration. (F) Proliferation assay of A549 cells treated with canagliflozin (5–30 μm) and either negative control siRNA (5 nm) or HIF‐1α siRNA (5 nm) for 5 days. (G) Representative protein immunoblots and (H) corresponding quantification of HIF‐1α from A549 cells treated with canagliflozin (30 μm), Roxadustat (30 μm) or a combination treatment for 48 h. All western blot data were normalized to β‐actin. (I) Proliferation assay of A549 cells treated with canagliflozin (30 μm), Roxadustat (30 μm), or combination treatment with or without RT (single fraction of 2 Gy 5 h after drug treatment initiation). RNA‐seq data were obtained from one experiment with three technical replicates. Protein immunoblotting and proliferation experiments were obtained from three independent experiments and are presented as mean ± SEM. Two‐way ANOVA (C, F) or ordinary one‐way ANOVA (E, H, I) followed by Tukey's multiple comparisons test was used to determine statistical significance. ns: nonsignificant, *P < 0.05, **P < 0.01, ***P < 0.001, ****P < 0.0001.

The heatmap in Fig. 7A shows the genes downregulated by canagliflozin involved in HIF‐1α signaling, including effectors that regulate HIF‐1α stability and nuclear transfer (*EGLN1*, *EGLN3*, *P4HA1*, and *P4HA2*), glucose and lipid metabolism (*SCD*, *ALDOA*, *LDHA*, *SLC2A3*, *GBE1*, *PFKFB3, PGK1, PLIN2*, and *HILPDA*), tyrosine kinase receptor signaling (*FGFR1*), protein kinase A signaling (*AKAP12*), apoptosis (*BIRC5*, *BNIP3*, *BNIP3L*, *FAM162A*, and *NDRG1*), histone deacetylation and demethylation (*HDAC2* and *KDM3A*), extracellular matrix regulation (*TFPI2*, *ANGPTL4*, and *PLOD2*), and iron ion cell homeostasis (*HMOX1*, *CP*, and *TFRC*).

Importantly, canagliflozin suppressed HIF‐1α protein levels in nonirradiated and irradiated A549 cells grown in standard normoxic cultures (Fig. 7B,C). To determine whether HIF‐1α suppression contributes to canagliflozin's antiproliferative potential, we performed HIF‐1α knockdown experiments with small interfering RNA (siRNA; Fig. 7D,E). Suppression of HIF‐1α expression led to improved antiproliferative response to canagliflozin treatment (Fig. 7F). Furthermore, cell treatment with the HIF‐prolyl hydroxylase inhibitor, Roxadustat (FG‐4592), suppressed HIF‐1α degradation (Fig. 7G,H) and provided partial rescue from the antiproliferative effects of canagliflozin (30 μm) alone and combined with RT (2 Gy; Fig. 7I). These results support the notion that HIF‐1α suppression is an important component of canagliflozin's mechanism of action.

### 3.3 Transcriptional regulation

**Fig. 8:**
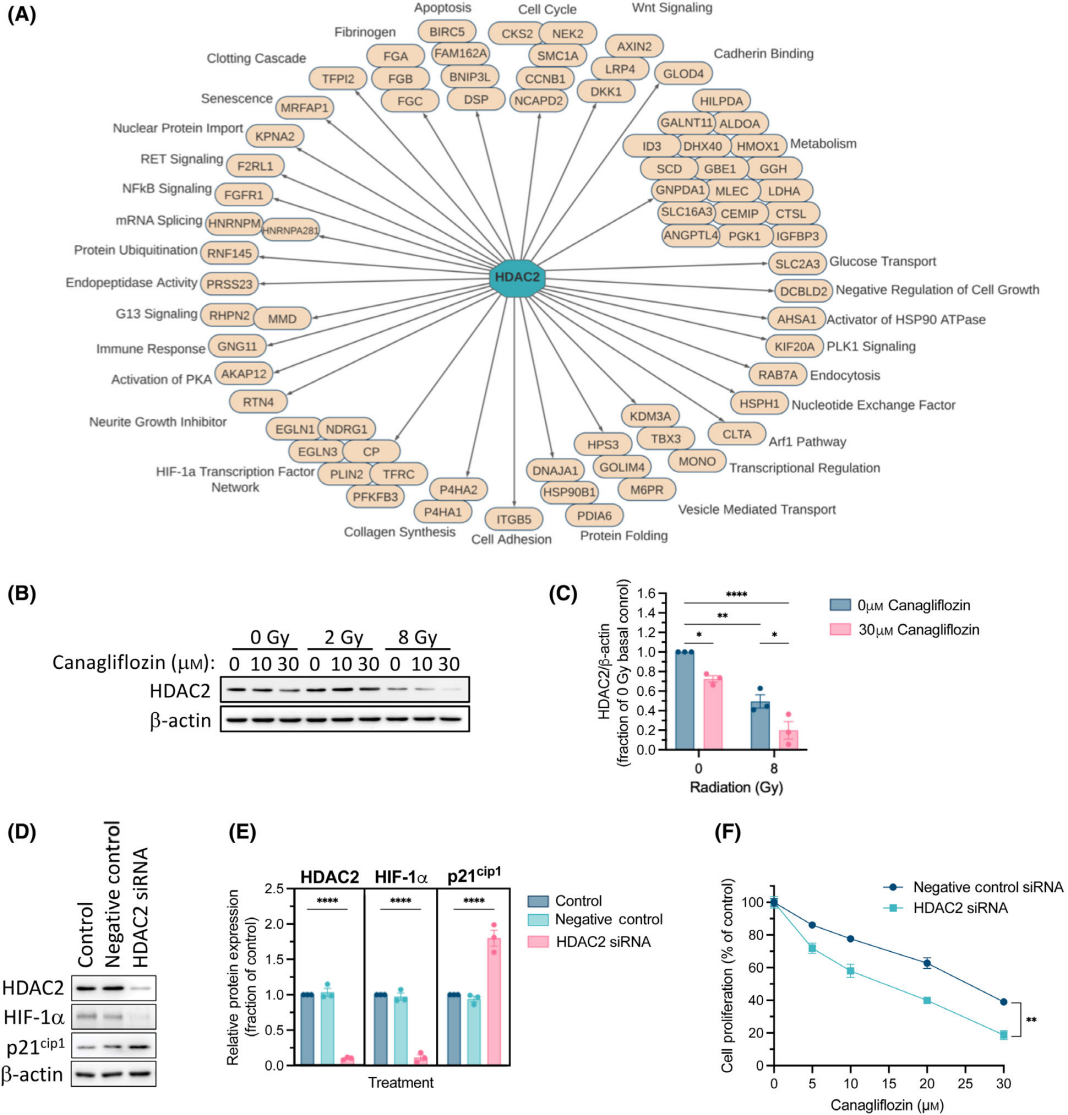
Regulatory network analysis and identification of HDAC2 as the modulator of canagliflozin's gene expression profile. (A) All downregulated DEGs; FDR *q*‐value < 0.05 from the RNA‐seq data were analyzed using Cytoscape's iRegulon plugin to predict the master regulators that mediate canagliflozin's transcriptional program. HDAC2, which was suppressed by canagliflozin, was identified as the key mediator associated with the drug's gene expression profile. Gene regulatory network analysis illustrates the HDAC2‐regulated genes that were significantly downregulated by canagliflozin in the RNA‐seq dataset (Normalized Enrichment Score = 3.66; FDR *q*‐value = 0.022). (B) Representative protein immunoblot and (C) quantification of HDAC2 in A549 cells pretreated with canagliflozin (10 or 30 μm) for 5 h and subsequently treated with RT (single fraction of 2 or 8 Gy) and analyzed 48 h later. (D) Representative protein immunoblots and of HDAC2, HIF‐1α, and p21^cip1^ in A549 cells treated with negative control siRNA (5 nm) or HDAC2 siRNA (5 nm) for 72 h (quantification of in (E)). All western blot data were normalized to β‐actin. (F) Proliferation assay of A549 cells treated with canagliflozin (5–30 μm) and either negative control siRNA (5 nm) or HDAC2 siRNA (5 nm) for 5 days. Results were derived from three independent experiments and presented as mean ± SEM. Ordinary one‐way ANOVA (E) or two‐way ANOVA (C, F) followed by Tukey's multiple comparisons test was used to determine statistical significance. *P < 0.05, **P < 0.01, ****P < 0.0001.

To help understand the processes underlying canagliflozin's transcriptional reprogramming, we implemented gene regulatory network analysis with Cytoscape's iRegulon tool. Consistent with the observed suppression of the *HDAC2* gene itself (Fig. 8A), iRegulon analysis determined HDAC2 as the transcriptional regulator responsible for canagliflozin's reprogramming [false discovery rate (FDR) *q*‐value = 0.022]. Fig. 8A illustrates the wide variety of HDAC2‐regulated biological processes we found to be downregulated by a clinically achievable concentration of canagliflozin in NSCLC cells.

Canagliflozin (30 μm) suppressed HDAC2 protein expression and radiation (8 Gy) provided further suppression in A549 cells (Fig. 8B,C). The finding that HDAC2 was suppressed by 8Gy alone is consistent with *HDAC2* downregulation detected in the RNA‐seq data of A549 cells treated with 5 Gy (Fig. S7A). To verify that HDAC2 is indeed a mediator of canagliflozin's antiproliferative effects, we knocked down HDAC2 with siRNA in A549 cells. siRNA‐mediated knockdown of HDAC2 downregulated HIF‐1α but increased p21^cip1^ expression in A549 cells (Fig. 8D,E). Importantly, HDAC2 knockdown improved the antiproliferative capacity of canagliflozin in A549 cells (Fig. 8F).

### 3.4 Canagliflozin target genes are associated with poor prognosis in NSCLC

**Fig. 9:**
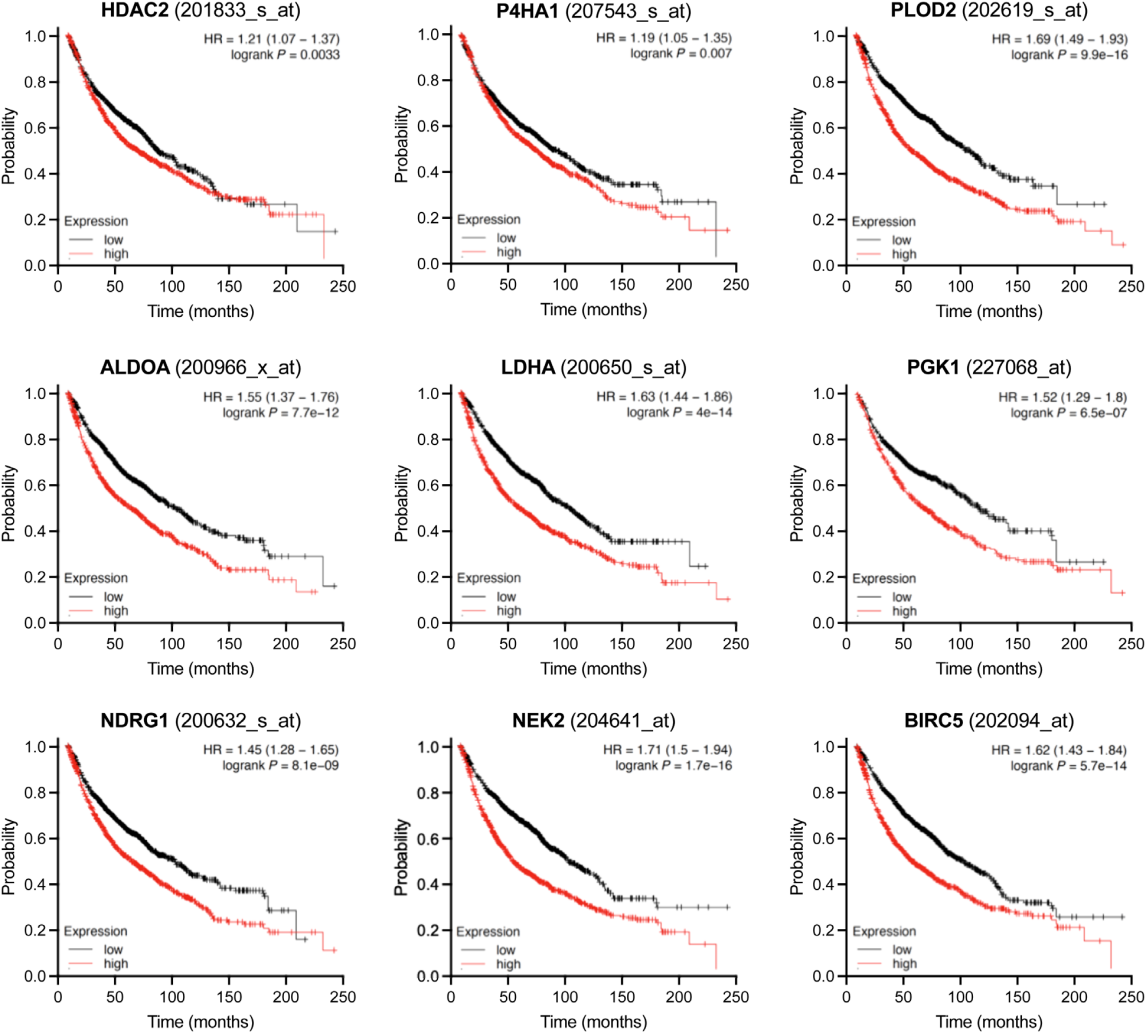
Prognostic value of canagliflozin‐regulated genes. The Kaplan–Meier Plotter database (kmplot.com) was used to generate Kaplan–Meier survival curves corroborating the association of the clinical outcome of key significantly downregulated genes (*HDAC2*, *P4HA1*, *PLOD2*, *ALDOA*, *LDHA*, *PGK1*, *NDRG1*, *NEK2*, and *BIRC5*) by canagliflozin in A549 cells. Datasets used for this analysis were from the following trials: GSE14814, GSE19188, GSE29013, GSE30219, GSE31210, GSE3141, GSE1908, GSE37745, GSE43580, GSE4573, GSE50081, and GSE8894.

The Kaplan–Meier Plotter database was employed to evaluate the prognostic value of DEGs targeted by canagliflozin in NSCLC datasets [37]. The database is generated from the Cancer Biomedical Informatics Grid, the Gene Expression Omnibus, and The Cancer Genome Atlas cancer datasets and includes NSCLC patients at different stages with various histologies and treatment regimens (see Fig. 9 legend for trial datasets included) [37]. Interestingly, results revealed that higher expression of several genes downregulated by canagliflozin is associated with poor prognosis of NSCLC. This included genes involved in transcription (*HDAC2*), hypoxia response and extracellular matrix synthesis (*P4HA1*, *PLOD2*), glucose metabolism (*ALDOA*, *LDHA*, and *PGK1*), growth factor response, proliferation, and prevention of apoptosis (*NDRG1*, *NEK2*, and *BIRC5*; Fig. 9).

## 4 Discussion

Resistance of tumor cells to cytotoxic therapy presents a significant obstacle to successful treatment. Mounting evidence suggests that metabolic reprogramming through activation of the PI3K‐Akt–mTOR pathway is a principal mechanism of resistance [42]. Previously, we showed that canagliflozin blocks mitochondrial complex‐I‐supported respiration leading to AMPK activation and suppression of the mTOR pathway in cancer cells [19]. Although SGLT2 is expressed in NSCLC cells (see Fig. S8) and canagliflozin inhibits glucose uptake in cancer cells, in that study, we found that the drug's anticancer action was independent of the extracellular glucose concentration [19]. Moreover, pyruvate or acetate supplementation in cell culture media did not reverse canagliflozin's antiproliferative activity (see Supplemental Data in Villani *et al*. [19]). Papadopoli *et al*. [43] also found that canagliflozin's antitumor activity is through SGLT2‐independent mechanisms, as canagliflozin's effects occurred in the absence of glucose and with knockdown of SGLT2 in breast cancer cells. Furthermore, we showed that canagliflozin's action was partially inhibited by overexpression of the biguanide‐resistant *Saccharomyces cerevisiae* complex‐I, NADH dehydrogenase 1 (ND1) [19]. These off‐target findings have since been corroborated by others [44]. Collectively, these data indicate that canagliflozin's antitumor activity does not rely upon reduced carbohydrate availability to cancer cells but relies, at least in part, on mitochondrial OxPhos complex‐I inhibition.

The primary objectives of the present study were to (1) investigate whether canagliflozin can further enhance the efficacy of cytotoxic therapy in preclinical models of NSCLC and (2) gain additional insights into the molecular mechanisms involved.

Here, we report that canagliflozin effectively suppressed NSCLC cell proliferation and survival and potentiated the efficacy of RT *in vitro*. Additive and synergistic effects were detected with clinically achievable canagliflozin and RT doses, an effect observed with even lower drug doses in clonogenic assays (Fig. 1). Importantly, the latter results were reproduced in three xenograft models (Fig. 2). Given that the purpose of this study was to focus on the efficacy of clinically achievable doses of canagliflozin and to investigate the combined therapy, we refrained from using high doses of cytotoxic therapy. Therefore, it is not unexpected that neither canagliflozin nor RT generated statistically significant antitumor efficacy as single agents, but combined therapy mediated this effect.

*In vivo* studies in other cancer types have reported significant suppression of tumor growth and prolonged survival with oral gavage of canagliflozin (10–300 mg·kg^−1^·day^−1^) [22, 24, 25]. In humans, treatment with 100 and 300 mg canagliflozin tablets provides plasma doses of 1.4–4.2 mg·kg^−1^·day^−1^ and *C*_max_ levels of 1096–4678 ng·mL^−1^ (2.5–10.5 μm), while intravenous infusion is well‐tolerated up to 38 μm [45, 46, 47]. Miller *et al*. [48] treated mice long term with 30 mg·kg^−1^·day^−1^ of canagliflozin mixed in diet, which yielded plasma concentrations of 360–860 ng·mL^−1^ (0.8–1.9 μm). This dosing is similar to a 50 mg canagliflozin dose in humans (*C*_max_ = 426–536 ng·mL^−1^; 0.9–1.2 μm) [46]. Therefore, based on these findings, we generated a canagliflozin diet to deliver 60 mg·kg^−1^·day^−1^ (resulted in about 55–80 mg·kg^−1^·day^−1^ in our cohorts) to mimic the plasma bioavailability of a human taking 100 or 300 mg canagliflozin tablets. This treatment improved the RT response and repressed tumor growth in all NSCLC xenografts (Fig. 2B).

We observed that a clinically relevant dose of canagliflozin (10 μm) rapidly (24 h) mediated reprogramming of NSCLC transcriptional activity leading to a gene expression profile consistent with suppression of growth factor signaling, cell cycle progression, and response to hypoxia, the latter of which induces glycolytic gene expression, survival, and radioresistance (Fig. 3). Therapeutically relevant canagliflozin doses suppressed mitochondrial respiration in NSCLC cells (Fig. 4), which triggered an expected induction of electron transport chain genes (Fig. 3C).

Compared with the selective mitochondrial complex‐I inhibitors, BAY‐87‐2243 and IACS‐010759, canagliflozin mediated moderate metabolic stress, reduced rates of extracellular acidification and increased antiproliferative activity (Fig. 4D–H). This was consistent with suppression of glycolytic genes such as aldolase A (*ALDOA*), phosphoglycerate kinase 1 (*PGK1*), 6‐phosphofructo‐2‐kinase/fructose‐2,6‐biphosphatase 3 (*PFKFB3*), lactate dehydrogenase A (*LDHA*; Fig. 7A), as well as *SLC16A3*, a gene encoding for monocarboxylic acid transporter 3, which transports pyruvate and lactic acid across membranes (Figs 3B and 8A; Fig. S7). The sustained metabolic stress after treatment with the OxPhos inhibitors (BAY‐87‐2243 and IACS‐010759) suggests that they irreversibly blocked mitochondrial complex‐I. A possible explanation is that the incomplete electron transfer in the mitochondria due to complex‐I blockade generated free radicals, which damaged mitochondrial components, including inactivating complex‐I activity [49]. The cells metabolically switched toward a glycolytic phenotype to maintain energy levels as indicated by elevated ECAR (Fig. 4D–H). These findings highlight an antitumor activity of canagliflozin with a reduced risk for extracellular acidification and lactic acidosis. It indicates a significant advantage for canagliflozin over selective and potent mitochondrial OxPhos inhibitors, which have failed to show benefit in clinical trials [50, 51].

Blockade of mitochondrial metabolism is intimately linked with AMPK activation and suppression of *de novo* lipogenesis, protein synthesis, and cell proliferation [7]. We found that canagliflozin mediated activation of AMPK, inhibition of ACC and the mTOR‐HIF‐1α pathway, and an associated suppression of the mitosis marker, p‐H3^Ser10^, in NSCLC cells. Similar results were obtained in other cancer models [22, 25]. In irradiated cells, canagliflozin amplified these effects, providing increased suppression of mTOR and its effectors. Notably, monotherapy with RT (5Gy) and oral canagliflozin induced sustained activation of the AMPK‐ACC pathway and reduced p‐H3^Ser10^ in tumors, and these effects were enhanced with the combined treatment (Fig. 5I,J). The latter was coupled with the inhibition of activating ERK1/2 phosphorylation (Thr202/204) in nonirradiated and irradiated NSCLC cells (Fig. 5H). These data suggest that: (1) continuous oral delivery of canagliflozin mediates activation of AMPK, suppression of growth factor signaling and metabolic stress response in tumors, (2) RT also induces sustained elevation of baseline AMPK activity, and (3) the combined treatment further enhances these events in surviving tumor cells.

Our results suggest that canagliflozin treatment alone triggered G_1_/S phase arrest (Fig. 6A), which is in agreement with previous reports [52, 53]. Canagliflozin rapidly upregulated transcripts of the cell cycle regulation genes, *TP53*, *CDKN1A*, and *CDKN1B* at 2 h (Fig. 6B). Importantly, in irradiated cells, canagliflozin treatment led to enhancement of the RT‐induced G_2_/M checkpoint and reduction of cells in G_1_ and S phases. The G_2_/M phase is regarded as the most radiosensitive stage of the cell cycle [54]. Therefore, this effect can be biologically important in the setting of fractionated RT where RT fractions are delivered daily or every 2–3 days. Our analysis of tumor death mechanisms suggested a detectable increase in apoptosis with canagliflozin, RT, and the combined treatment but no clear evidence of increased necrosis (Fig. 5; Fig. S5). The lack of extensive evidence of cell death in tumors weeks after cytotoxic therapy is not unexpected. The sustained improved suppression of growth in tumors receiving combined treatments may result from initial enhanced cell death with combined drug and RT treatment and continued cytostatic effects of canagliflozin in the long term.

HIF‐1α is a key driver of glucose metabolism that promotes cell survival, migration, and angiogenesis, all of which contribute to tumor resistance to cytotoxic therapy [55]. The work presented here shows suppression of HIF‐1α activity by canagliflozin. Our RNA‐seq data underlined the importance of canagliflozin‐mediated downregulation of the HIF‐1α pathway. While we did not find *HIF1A* to be among the downregulated DEGs, its regulation by canagliflozin may be post‐translational. The results of HIF‐1α knockdown or pharmacological stabilization validated the notion of HIF‐1α suppression as a critical mechanism for canagliflozin's activity (Fig. 7). It appears that loss of HIF‐1α activity generates a vulnerability to metabolic stress induced by canagliflozin suggesting generation of a positive feedback loop whereby canagliflozin mediates sensitivity to its own effects and those of RT. A study by Faubert *et al*. [56] using A549 cells suggested that loss of the tumor suppressor, liver kinase B1 (LKB1), leads to metabolic reprogramming mediated by HIF‐1α that involves increased expression of genes like *ALDOA*, *PDK1*, and *LDHA* and increased glucose and glutamine metabolism through the tricarboxylic acid (TCA) cycle. They postulated that HIF‐1α targeting would generate an opportunity for synthetic lethality in LKB1‐deficient tumors. Nevertheless, in this study, we observed effective suppression of HIF‐1α, proliferation, and tumor growth in both LKB1‐deficient (A549) and LKB1‐proficient (H1299) NSCLC cells and tumors (Fig. 7B,C; Fig. S4B). These findings support a strong potential for canagliflozin to reprogram tumor metabolism, inhibit NSCLC survival, and enhance the effects of cytotoxic therapy.

Our transcriptional regulatory network analysis identified *HDAC2* as the putative gene mediating canagliflozin's transcriptional program (Fig. 8A). Histone deacetylases remove acetyl groups and are markers of epigenetic repression. They play global roles in regulating gene transcription, proliferation, and survival but are also notoriously overexpressed in solid malignancies [57]. HDAC2 is shown to inhibit cell cycle checkpoints through suppression of genes like *CDKN1A* (p21^cip1^) [58] and mediate cell migration and invasion through HIF‐1α stabilization [59]. Here, we found that canagliflozin and radiation suppress HDAC2 transcript (Fig. S7A) and the cellular HDAC2 protein levels while combined treatment does so more effectively (Fig. 8B,C). The results of our HDAC2 knockdown experiments (Fig. 8) demonstrated that HDAC2 controls cellular HIF‐1α and p21^cip1^ levels and confers resistance to canagliflozin's antiproliferative activity consistent with the regulation of HIF‐1α (Fig. 7). Recently, another study suggested that canagliflozin directly targets and suppresses HDAC6 in gastrointestinal cancer cells [60], indicating that HDAC targeting may be a common pathway for the activity of this drug in tumors.

Many of the genes that were significantly upregulated by RT (*FGFR1*, *ALDOA*, *RNF145*, *PGK1*, *P4HA1*, *PLOD2*, *IGFBP3*, *NDRG1*, *PFKFB3*, and *BNIP3L*) are associated with glycolysis, cell cycle progression, prevention of apoptosis, angiogenesis, cholesterol homeostasis, and survival, which contribute to resistance to cytotoxic therapy. In contrast, canagliflozin downregulated these genes, highlighting additional pathways for this drug to curtail radioresistance induced by RT (see Fig. S7A,B for a complete list of canagliflozin‐regulated DEGs and comparative effects of RT).

Studies with pancreatic and liver cancer xenograft models suggested that canagliflozin can improve chemotherapy responses [23, 61]. While the focus of this study was to investigate the combination of canagliflozin with RT for the treatment of NSCLC, we also found that canagliflozin improved the efficacy of the key chemotherapeutic agents of NSCLC, cisplatin, and etoposide, as well as concurrent chemo‐RT *in vitro* (Fig. S9). These findings lay out arguments for future clinical investigations of canagliflozin in advanced NSCLC where combined cytotoxic treatment with chemo‐RT is the standard of care.

Our findings that canagliflozin suppressed several genes involved in tumor survival, which hold poor prognostic value in clinical NSCLC datasets (Fig. 9), further strengthen the rationale for clinical investigation of this agent in NSCLC. However, beyond strengths shared with other agents with tumor‐suppressive and RT−/chemo‐sensitizing activities, canagliflozin holds a unique quality that may lead to an improved therapeutic ratio if combined with standard‐of‐care therapies for LA‐NSCLC. Clinical studies in the past 5 years show that unintentional delivery of RT to the heart during chest irradiation for LA‐NSCLC, which often cannot be avoided due to the anatomical presentation of tumors, leads to poor overall survival [62, 63]. Notably, based on phase III randomized trial evidence, canagliflozin is now also approved for the prevention and management of heart failure [64], indicating a potential for cardio‐protection. This notion and the lack of evidence associating canagliflozin with any toxicity in patients receiving cancer therapy provide additional rationale for canagliflozin to be investigated in LA‐NSCLC.

## 5 Conclusions

Our results show that the widely used SGLT2 inhibitor, canagliflozin, exerts tumor‐suppressive activity and enhances NSCLC response to standard cytotoxic therapy. Canagliflozin's antineoplastic effects are attributed, in part, to mitochondrial respiration blockade, inhibition of the mTOR‐HIF‐1α pathway, and substantial transcriptional reprogramming mediated likely through suppression of HDAC2. Canagliflozin is a well‐tolerated medication that is approved for the treatment of type 2 diabetes and heart failure. It is well tolerated by nondiabetics and there is no known increase in toxicity when combined with standard cytotoxic therapy, making it a favorable agent for clinical investigation in NSCLC.

## Conflict of interest

The authors declare no conflict of interest.

## Author contributions

All authors contributed to the publication according to the ICMJE guidelines for authorship. All authors agreed to be accountable for all aspects of the work in ensuring that questions related to the accuracy or integrity of any part of the work are appropriately investigated and resolved. ODB and TT contributed to study concept and design and drafting of the manuscript. ODB, EET, AA, EA, JW, SW, BM, KS, GM, and TF contributed to data acquisition. ODB, EET, AA, EA, JW, SW, BM, KS, BA, RKS, AM, PE, TB, JLB, PM, GRS, and TT contributed to analysis and interpretation of data. ODB and EET contributed to statistical analysis. ODB, EET, AA, EA, JW, SW, BM, KS, GM, TF, BA, RKS, AM, PE, TB, JLB, PM, GRS, and TT contributed to manuscript review and approval. GRS and TT obtained funding.

### Peer review

The peer review history for this article is available at https://www.webofscience.com/api/gateway/wos/peer‐review/10.1002/1878‐0261.13508.

## Supporting information


**Fig. S1.** Body weight and food and water intake of mice bearing non‐small cell lung cancer (NSCLC) tumors.
**Fig. S2.** Bioinformatics analysis of differentially expressed genes (DEGs) in A549 non‐small cell lung cancer (NSCLC) cells treated with canagliflozin or radiotherapy (RT).
**Fig. S3.** Effects of canagliflozin and radiotherapy (RT) on mitochondrial respiration in A549 non‐small cell lung cancer (NSCLC) cells.
**Fig. S4.** Regulation of metabolic pathways by canagliflozin and radiotherapy (RT) in H1299 non‐small cell lung cancer (NSCLC) cells.
**Fig. S5.** Assessment of necrosis in H1299 non‐small cell lung cancer (NSCLC) tumors.
**Fig. S6.** Analysis of cell cycle regulation genes in A549 non‐small cell lung cancer (NSCLC) cells treated with canagliflozin.
**Fig. S7.** Differentially expressed genes (DEGs) in A549 non‐small cell lung cancer (NSCLC) cells treated with canagliflozin or radiotherapy (RT).
**Fig. S8.** Expression of glucose transporters in non‐small cell lung cancer (NSCLC) cells.
**Fig. S9.** Proliferation assay of human non‐small cell lung cancer (NSCLC) cells treated with canagliflozin, chemotherapy, and/or radiotherapy (RT).
**Table S1.** List of antibodies and PCR probes used.

## Data Availability

RNA‐seq data that support the findings in this study are openly available in NCBI's Gene Expression Omnibus and are accessible through https://www.ncbi.nlm.nih.gov/geo/query/acc.cgi?acc=GSE239495, GEO Series accession number [GSE239495]. All other data are available from the corresponding author upon reasonable request.
