# Supplementary material for: Canagliflozin mediates tumor suppression alone and in combination with radiotherapy in non‐small cell lung cancer (NSCLC) through inhibition of HIF‐1α
Source: Mol Oncol. 2023 Aug 27;17(11):2235–56. doi: 10.1002/1878-0261.13508 (PMC10620129; doi:10.1002/1878-0261.13508)
Supplement: Supplementary file 1 — Fig. S1. Body weight and food and water intake of mice bearing non‐small cell lung cancer (NSCLC) tumors. Fig. S2. Bioinformatics analysis of differentially expressed genes (DEGs) in A549 non‐small cell lung cancer (NSCLC) cells treated with canagliflozin or radiotherapy (RT). Fig. S3. Effects of canagliflozin and radiotherapy (RT) on mitochondrial respiration in A549 non‐small cell lung cancer (NSCLC) cells. Fig. S4. Regulation of metabolic pathways by canagliflozin and radiotherapy (RT) in H1299 non‐small cell lung cancer (NSCLC) cells. Fig. S5. Assessment of necrosis in H1299 non‐small cell lung cancer (NSCLC) tumors. Fig. S6. Analysis of cell cycle regulation genes in A549 non‐small cell lung cancer (NSCLC) cells treated with canagliflozin. Fig. S7. Differentially expressed genes (DEGs) in A549 non‐small cell lung cancer (NSCLC) cells treated with canagliflozin or radiotherapy (RT). Fig. S8. Expression of glucose transporters in non‐small cell lung cancer (NSCLC) cells. Fig. S9. Proliferation assay of human non‐small cell lung cancer (NSCLC) cells treated with canagliflozin, chemotherapy, and/or radiotherapy (RT). Table S1. List of antibodies and PCR probes used. [file MOL2-17-2235-s001.pdf]

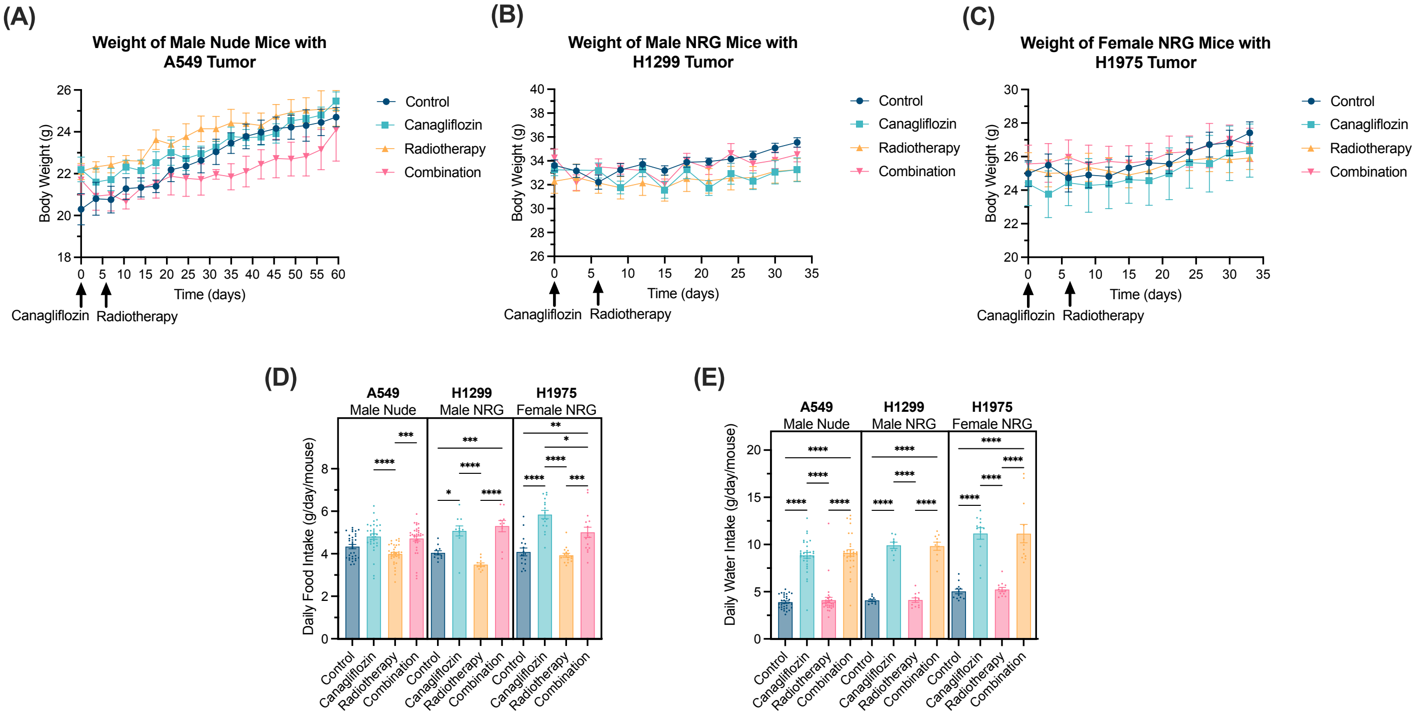

Supplementary Figure 1. Body weight and food and water intake of mice bearing non-small cell lung cancer (NSCLC) tumors. Total body weight of **(A)** male BALB/c nude mice with A549 tumors (*n*=4–5/group), **(B)** male NRG mice with H1299 tumors (*n*=5/group) and **(C)** female NRG mice with H1975 tumors (*n*=5/group) treated with vehicle control, canagliflozin, RT (5Gy) or a combination treatment. **(D)** Diet and **(E)** water were weighed 2–3 times per week to estimate the daily consumption by the animals. Data are presented as mean ± SEM. **(A–C)** Two-way repeated measures ANOVA or **(D, E)** ordinary one-way ANOVA followed by Tukey's multiple comparisons test was used. *$p<0.05$, **$p<0.01$, ***$p<0.001$, ****$p<0.0001$.

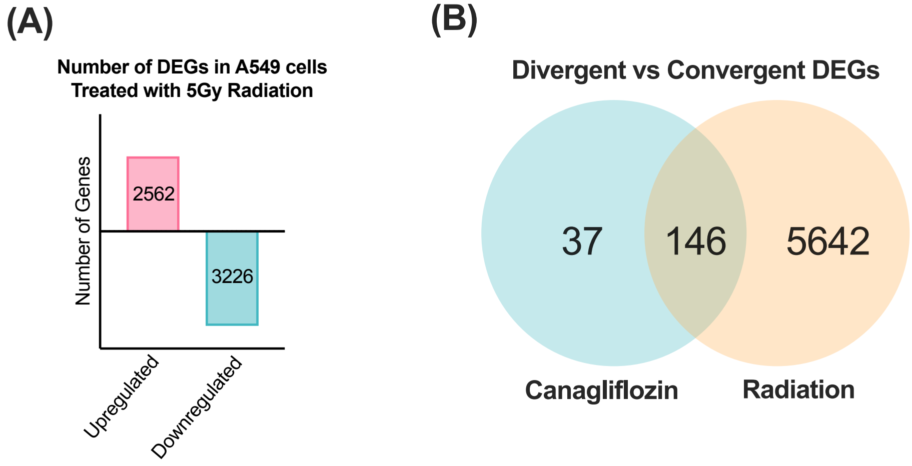

Supplementary Figure 2. Bioinformatics analysis of differentially expressed genes (DEGs) in A549 non-small cell lung cancer (NSCLC) cells treated with canagliflozin or radiotherapy (RT). RNA-sequencing (seq) was used to provide insight into the transcriptome of A549 cells treated with 10μM canagliflozin or 5Gy for 24h (*n*=3). **(A)** The number of significantly upregulated and downregulated DEGs in A549 cells following RT (5Gy) with a False Discovery Rate (FDR) *q*-value <0.05. **(B)** A Venn diagram illustrating the number of divergent and convergent DEGs by canagliflozin (10μM) vs. RT (5Gy) (FDR *q*-value <0.05).

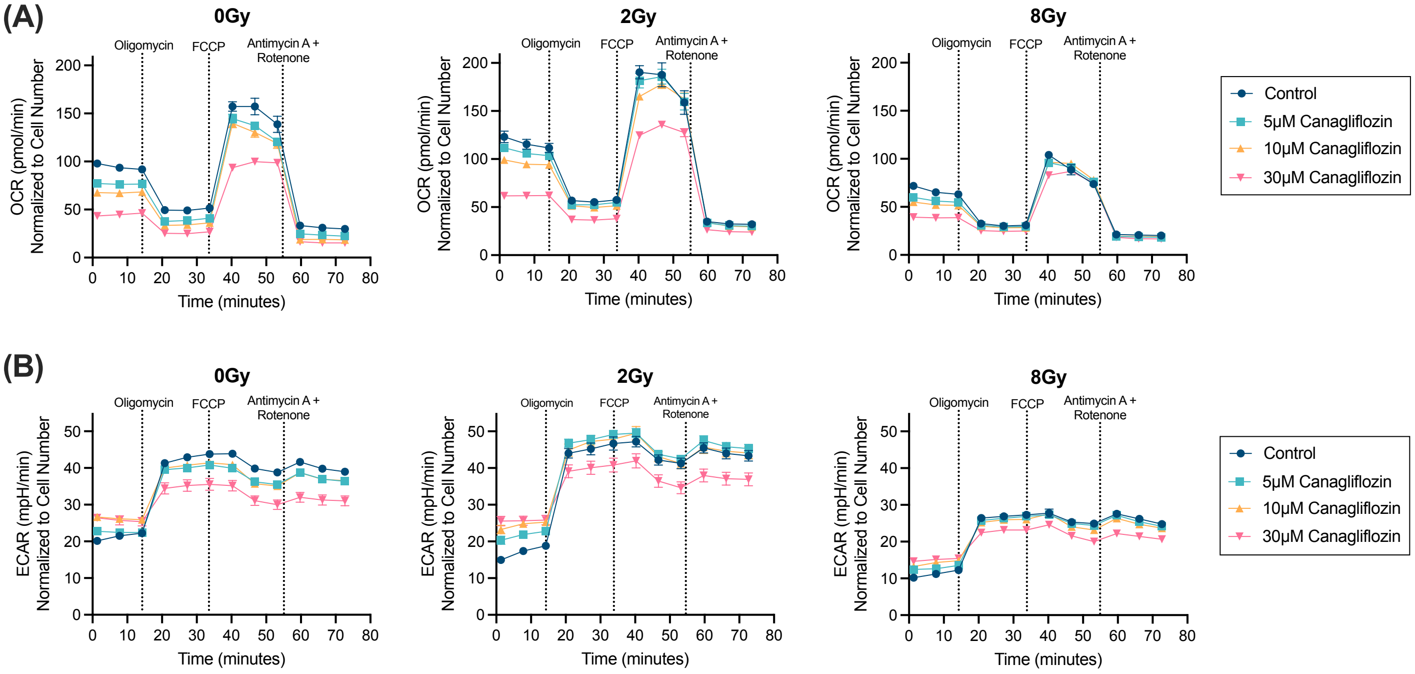

Supplementary Figure 3. Effects of canagliflozin and radiotherapy (RT) on mitochondrial respiration in A549 non-small cell lung cancer (NSCLC) cells. A549 cells were pre-treated for 5h with the indicated canagliflozin (5–30µM) doses prior to RT (single fraction of 2 or 8Gy) and **(A)** oxygen consumption rate (OCR) and **(B)** extracellular acidification rate (ECAR) were measured with the Seahorse Mito Stress Test 48h later.

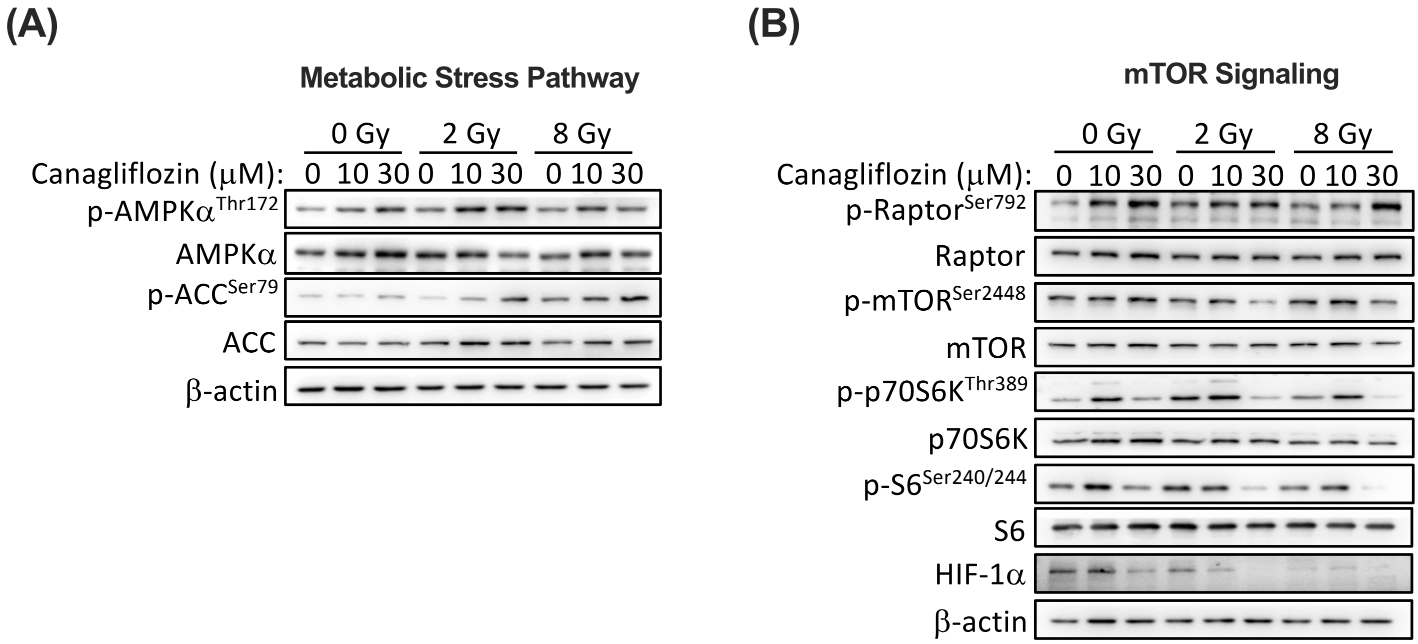

Supplementary Figure 4. Regulation of metabolic pathways by canagliflozin and radiotherapy (RT) in H1299 non-small cell lung cancer (NSCLC) cells. Representative protein immunoblots of H1299 cells pre-treated with canagliflozin (10 or 30µM) for 5h followed by RT (2 or 8Gy) and probed for markers in the **(A)** metabolic stress and **(B)** mTOR signaling pathways 48h post-RT.

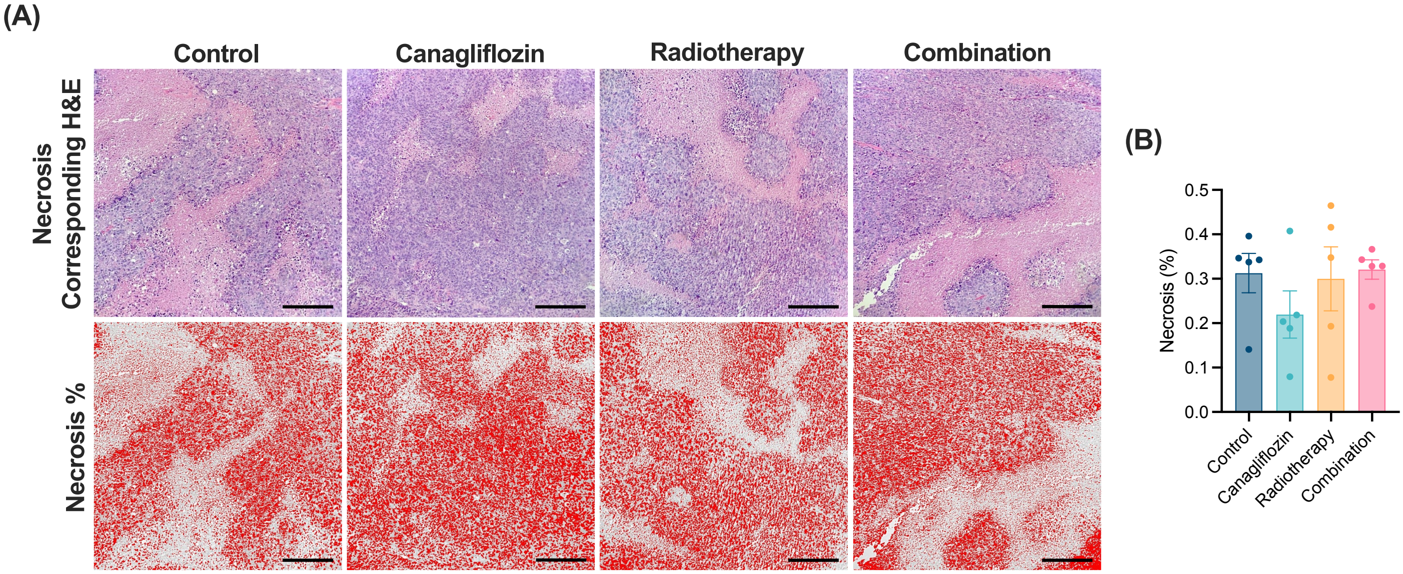

Supplementary Figure 5. Assessment of necrosis in H1299 non-small cell lung cancer (NSCLC) tumors. (A) Representative H&E images (top row) of H1299 tumors (*n*=5/group) that were treated with vehicle control, canagliflozin, radiotherapy or combination therapy and the necrosis percentage images (second row) were generated using ImageJ (scale bar=400μm). Red indicates the viable areas, while the pale color represents necrotic areas. **(B)** Quantification of the whole tumor section was determined following the ImageJ User Guide for tissue quantification. Necrotic Area = Total Area – Viable Tissue Area; Necrotic Tissue Percentage = Necrotic Area/Total Area. Data are presented as mean ± SEM. Ordinary one-way ANOVA **(B)** was used to detect statistical significance.

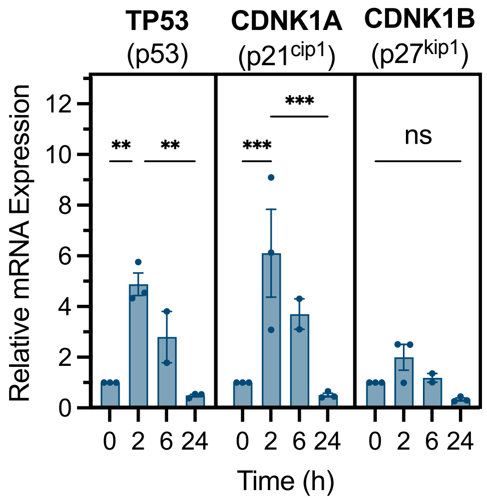

Supplementary Figure 6. Analysis of cell cycle regulation genes in A549 non-small cell lung cancer (NSCLC) cells treated with canagliflozin. Relative mRNA expression, determined with RT-qPCR, of cell cycle regulation genes in A549 cells treated with canagliflozin (30μM) for 0, 2, 6 and 24h. Data were obtained from three independent experiments and presented as mean ± SEM. Ordinary one-way ANOVA followed by Tukey's multiple comparisons test was used to detect statistical significance. ns: non-significant, ***p*<0.01, ****p*<0.001.

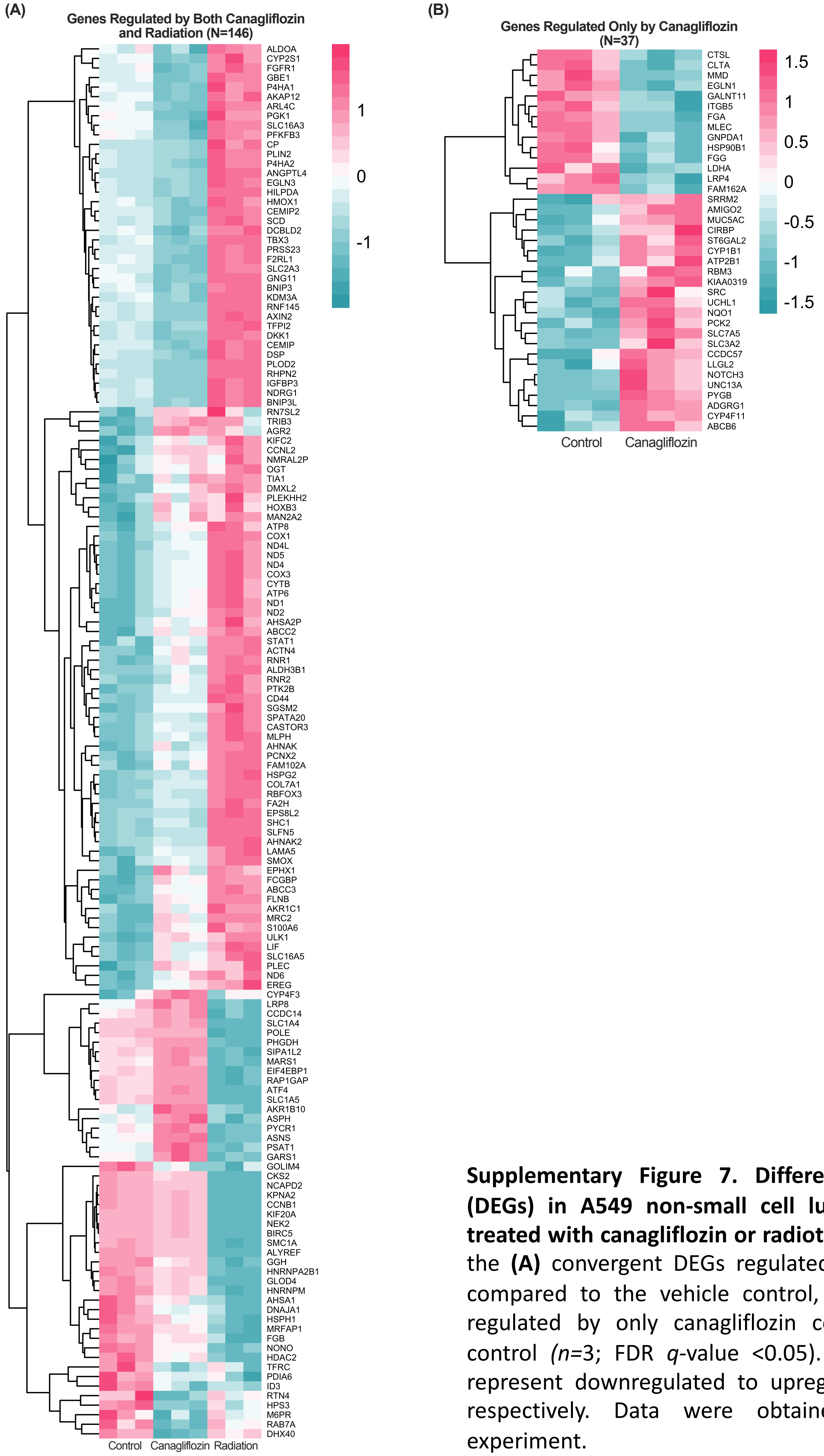

Supplementary Figure 7. Differentially expressed genes (DEGs) in A549 non-small cell lung cancer (NSCLC) cells treated with canagliflozin or radiotherapy (RT). Heatmaps of the **(A)** convergent DEGs regulated by canagliflozin and RT compared to the vehicle control, and **(B)** divergent DEGs regulated by only canagliflozin compared to the vehicle control (*n*=3; FDR *q*-value <0.05). The teal to pink scales represent downregulated to upregulated from -1.5 to 1.5, respectively. Data were obtained from the RNA-seq experiment.

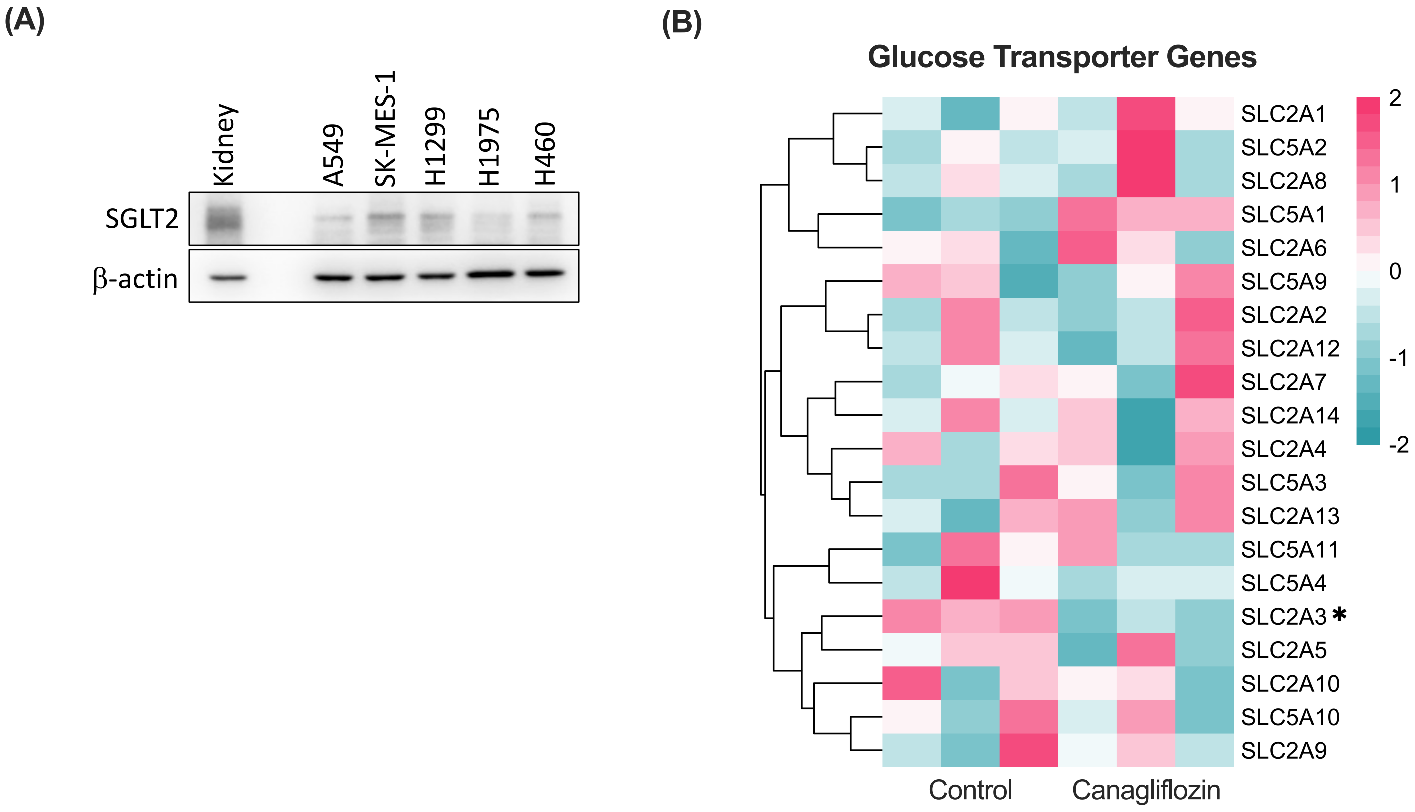

Supplementary Figure 8. Expression of glucose transporters in non-small cell lung cancer (NSCLC) cells. (A) Representative protein immunoblot of the sodium-glucose cotransporter 2 (SGLT2) in mouse kidney (positive control) and human A549, SK-MES-1, H1299, H1975 and H460 NSCLC cells. **(B)** A heatmap of glucose transporter genes from the RNA-seq data in A549 cells (control vs. canagliflozin). A 24h treatment with 10μM canagliflozin significantly regulated one glucose transporter (*SLC2A3*) as indicated by an asterisk (*) (false discovery rate (FDR) *q*-value <0.05).

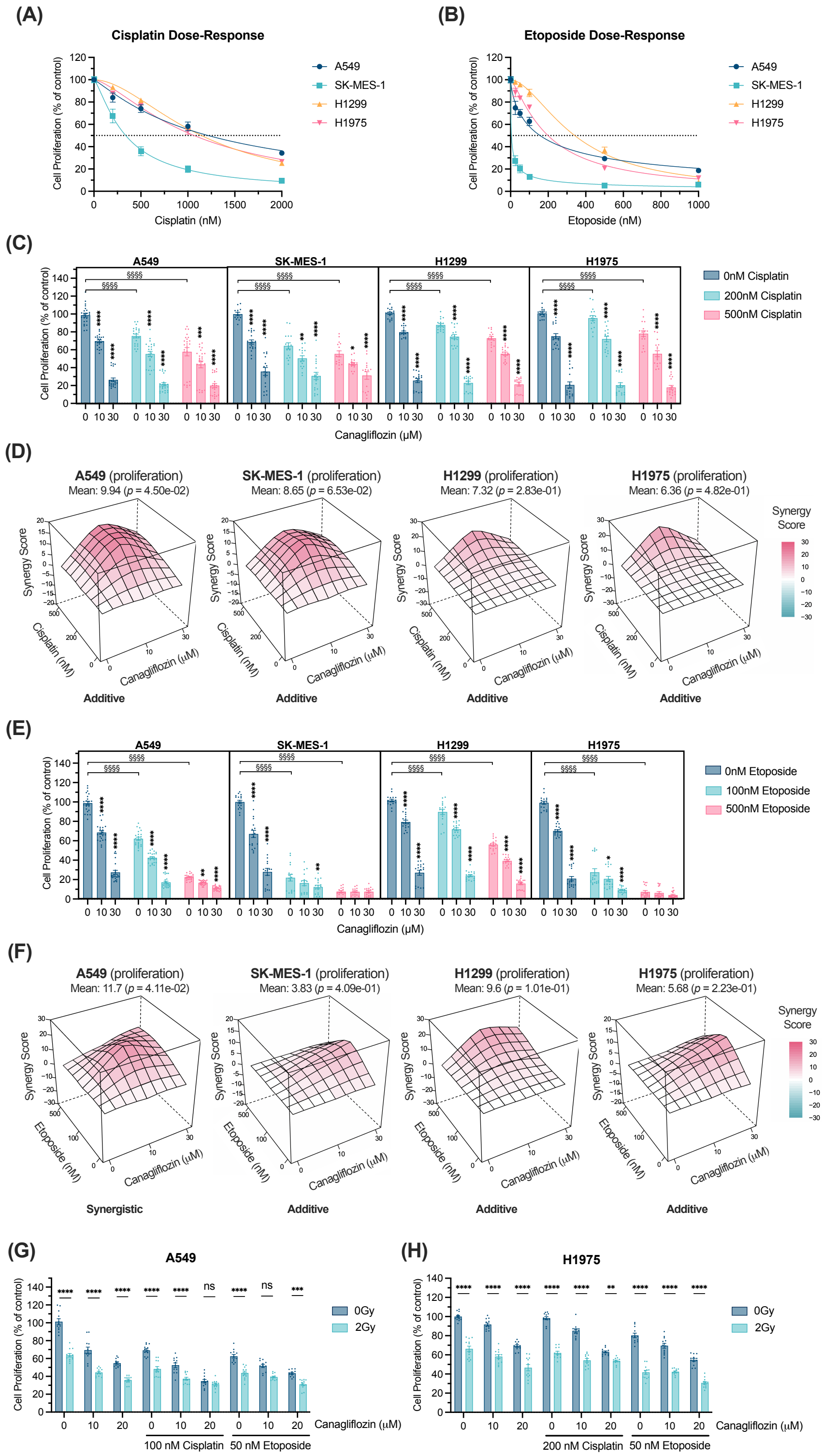

Supplementary Figure 9. Proliferation assay of human non-small cell lung cancer (NSCLC) cells treated with canagliflozin, chemotherapy and/or radiotherapy (RT). Proliferation assay of human NSCLC cells (A549, SK-MES-1, H1299 and H1975) treated with **(A)** cisplatin (200–2000nM), **(B)** etoposide (50–1000nM) or **(C, E)** a combination treatment concurrently with canagliflozin (10 or 30μM) for 5 days. **(D, F)** Proliferation assay results were subjected to synergy analysis using Highest Single Agent (HSA) modeling where a mean HSA synergy score of < -10 suggests antagonism, -10–10 suggests additivity and > 10 suggests synergism. Proliferation assay with **(G)** A549 and **(H)** H1975 cells treated with the indicated doses of canagliflozin (10 or 20μM), cisplatin (100 or 200nM), etoposide (50nM) and/or RT (single fraction of 2Gy). Drug treatments were administered concurrently 5h prior to RT. Results were derived from three independent experiments and presented as mean ± SEM. Data were calculated using two-way ANOVA **(C, E, G, H)** followed by Tukey's multiple comparisons test. ns: non-significant, *p<0.05, **p<0.01, ***/§§§p<0.001, ****/§§§§p<0.0001.

Supplementary Table 1. List of antibodies and PCR probes used. Antibodies were purchased from New England Biolabs (Ipswich, MA), Proteintech (Sankt Leon-Rot, Germany) or Vector Laboratories Inc (Burlingame, CA) and PCR probes were purchased from Life Technologies Inc (Carlsbad, CA).

Antibody or Probe	Catalog Number	Dilution (WB)	Dilution (IHC)	Manufacturer
AMPKα	2532	1:1000		NEB
p-AMPKα (Thr172)	2531	1:1000		NEB
ACC	3662	1:1000		NEB
p-ACC (Ser79)	11818	1:1000	1:200	NEB
Raptor	2280	1:1000		NEB
p-Raptor (Ser792)	2083	1:1000		NEB
mTOR	2983	1:1000		NEB
p-mTOR (Ser2448)	2971	1:1000		NEB
p70S6K	9202	1:1000		NEB
p-p70S6K (Thr389)	9205	1:1000		NEB
S6	2217	1:1000		NEB
p-S6 (Ser240/244)	2215	1:1000		NEB
4EBP1	9644	1:1000		NEB
p-4EBP1 (Ser65)	9451	1:1000		NEB
HIF-1α	36169	1:1000		NEB
H3	4499	1:1000		NEB
p-H3 (Ser10)	53348	1:1000		NEB
p21cip1	2947	1:1000		NEB
p27kip1	3688	1:1000		NEB
HDAC2	57156	1:1000		NEB
β-actin	5125	1:1000		NEB
SGLT2	24654-1-AP	1:1000		Proteintech
CC3 (Asp175)	9661		1:400	NEB
Anti-Rabbit IgG, HRP-linked secondary antibody	7074	1:10000		NEB
Goat anti-Rabbit IgG (H+L), Biotinylated	BA-1000		1:500	Vector Laboratories Inc
TP53 (p53)	4331182 (Hs01034249_m1)			Life Technologies Inc
CDKN1A (p21cip1)	4331182 (Hs00355782_m1)			Life Technologies Inc
CDKN1B (p27kip1)	4331182 (Hs00153277_m1)			Life Technologies Inc
GAPDH	4331182 (Hs02786624_g1)			Life Technologies Inc
